# Acute stress does not affect economic behavior in the experimental laboratory

**DOI:** 10.1371/journal.pone.0244881

**Published:** 2021-01-07

**Authors:** Róbert F. Veszteg, Kaori Yamakawa, Tetsuya Matsubayashi, Michiko Ueda

**Affiliations:** 1 School of Political Science and Economic, Waseda University, Tokyo, Japan; 2 School of Psychology, Tokai Gakuen University, Nagoya, Japan; 3 Osaka School of International Public Policy, Osaka University, Osaka, Japan; Universidad Loyola Andalucia Cordoba, SPAIN

## Abstract

We report statistical results from a laboratory experiment in which participants were required to make decisions with monetary consequences in several solitary and interactive situations under acute stress. Our study follows the tradition of behavioral and experimental economics in selecting the experimental situations and incorporates elements from medical and psychological research in the way stress is induced and measured. It relies on a larger sample, with 192 volunteers, than previous studies to achieve higher statistical power. The main conclusion, drawn from binary comparisons between the treatment and reference groups, is that acute stress does not have a significant impact on cognitive skills, strategic sophistication, risk attitudes, altruism, cooperativeness, or nastiness. Regression analysis, controlling for psycho-social characteristics, corroborates these findings, while also suggesting that acute stress significantly decreases men’s risk aversion (as measured by a lottery-choice risk-elicitation task).

## 1 Introduction

It is a commonplace that we live in a world full of stress. Many of our routine and even some of our major life decisions are made under circumstances that are typically assumed away by the canonical economic model of human decision-making. This study reports statistical results from a laboratory experiment in which participants were required to make decisions with monetary consequences in several solitary and interactive situations under acute stress. Our approach, particularly our situation list, follows the tradition of behavioral and experimental economics, while our experimental design and procedures incorporate elements from medical and psychological research.

A number of papers examining decision-making under stress have appeared over the last decade. One stream has appeared in medical journals and the other in scientific publications devoted to behavioral and social sciences, with considerable overlap due to coauthoring and shared methodology. The first (self-proclaimed) study of “the impact of stress on behavior in situations of strategic uncertainty” was carried out by Leder, Häusser and Mojzisch [1]. Following the medical tradition, they used a sample of 60 exclusively male participants, 31 of whom went through the group version of the so-called “Trier Social Stress Test” (TSST) used by the researchers to induce acute stress (see below for more on TSST). The authors used a multiplayer guessing game, a beauty-contest game, in four repetitions to measure strategic sophistication and conclude that stress results in less strategizing. These early results are based on a rather small sample. Moreover, a statistically significant difference between the treatment (TSST) and the benchmark groups appears only in the fourth round of interaction and after six observations are eliminated from the sample. Those six observations belong to participants who did not show the expected salivary cortisol-response after participating in the TSST.

In a later report, Leder, Häusser and Mojzisch [2] lend support to the earlier findings of a quasi-experiment in a real-world academic exam situation typically characterized by stress. In this new experiment, students waiting for an exam constitute the treatment group (and students waiting for a regular class form the reference group) and were asked to play the beauty-contest game. The results show that stress (caused by the exam) reduces strategizing and also impairs decision-makers’ correct understanding of the game.

Subsequent studies explored the effect of acute stress on risk attitudes, loss aversion, and consistency in decision-making. Sokol-Hessner et al. [3] find no evidence that acute stress affects behavior in decision-making about monetary earnings from risky lotteries, based on a sample of 120 participants assigned to the cold-pressor test or a control manipulation. However, Bendahan et al. [4] conclude that subjects with a cortisol-response to acute stress (induced by TSST) are more willing to take risks and are less strategic. They also find that stressed decision-makers are more self-oriented and less concerned about others’ monetary gains.

Following the methodological criticism of Taylor et al. [5] concerning the reliance on exclusively male participant pools, the literature targeted gender differences in how acute stress affects human decision-making. Kandasamy et al. [6] find that higher cortisol levels correlate with less risk-taking and that this effect is stronger for men than for women. The experimental design relied on administering hydrocortisone—the pharmaceutical form of cortisol—or a placebo to a relatively small number of participants (20 men and 16 women) in a double-blind setting. Similarly, Cahlíková and Cingl [7] conclude that acute stress significantly increases risk aversion in men when personal characteristics are controlled for, and has a similar but statistically insignificant impact on women. Their study collected data from a larger pool of participants (81 men and 70 women) and used the TSST for groups as a stressor, which was validated by three different measures (salivary cortisol levels, heart rate, and mood questionnaire scores). Regarding gender differences in behavior and characteristics other than risk attitudes under acute stress, Youssef et al. [8] measured cooperativeness using the ultimatum game in which females under stress seemed to be less likely to reject offers (even unfair ones) sent by a male proposer, while male participants’ behavior did not change across the stress-treatment and reference groups. Von Dawans et al. [9] find similar patterns using several binary-choice tasks designed to study pro-social behavior. They report increased levels of trustworthiness and willingness to share after stress in their exclusively female subject pool. These findings all seem to lend empirical support to the “tend-and-befriend” pattern hypothesized by Taylor et al. [5] for females under acute stress. For a brief survey on differing stress responses across gender groups, refer to Pruessner’s [10], who lists the possible biological and cultural causes.

The literature has also explored environments more relevant for economists and economics, such as highly competitive tournament settings [11, 12], markets with imperfect competition à la Cournot [13], decision-makers’ willingness to compete [14, 15], and economic rationality [16]. A similarly recent but much more extensive literature has appeared in economics related to happiness, which could be related to the above-reviewed research on acute stress. Lane [17] offers a small survey of the scientific reports on happiness and economic behavior. Among the correlates of happiness, he lists prosocial behavior and trust as positive factors and selfishness as a negative one. The reviewed literature does not deliver clear results on the relationship between happiness and risk preferences. Importantly, Lane [17] argues that “there is stronger evidence that the behavior is a cause of happiness than a consequence of it.” Regarding stress, it has been suggested that competition induces stress, as measured through participants’ heart rate and self-reports on subjective well-being [11], and that men consistently choke under pressure in high-stakes environments [12]. Cohen-Zada et al. [12] analyze data from real tennis tournaments with monetary rewards. Their study also considers female players, but the results are mixed. Buser et al. [14] conclude that stress (proxied by cortisol levels) is positively correlated with choosing to enter competitive environments for women but not for men. Contrariwise, Halko and Sääksvuori [15] argue that male participants with large acute heart-rate-variability response (to forced competition) tend to prefer tournaments, while this effect for female participants becomes statistically significant only after confidence and risk attitudes are controlled for. Concerning strategizing, Buckert et al. [13] found that acute stress results in more imitation (i.e., less deliberation and strategizing), which seems to be in line with the above-mentioned results showing less strategizing under stress in the beauty-contest game. However, economic rationality as measured through decision-makers’ consistency (and the adherence of their choices to the Generalized Axiom of Revealed Preference, [GARP]) does not seem to be affected by acute stress [16].

The literature review above suggests that we still lack a full understanding of how acute stress affects human behavior. With this paper, we contribute to the literature by reporting statistical results on human behavior under acute stress in a set of monetarily-incentivized decision problems that are considered standard and look very familiar to economists. Our goal is to test, in a carefully-controlled laboratory environment, the adequateness of the usual assumption in economic models according to which decision-making is unaffected by stress. We approach the problem with economic theory as our benchmark and test the effect of acute stress on a variety of outcomes, rather than on a single outcome as earlier studies have done, that are relevant for the economic model of decision-making. Importantly, some of the outcomes we study—such as altruism, cooperativeness, and nastiness—have never been studied with subjects under stress. We test cognitive abilities and strategic thinking using the “Cognitive Reflection Test” and a five-person beauty-contest game, and we measure risk attitudes in three different ways: with an open question, three lottery-choice decisions, and the bomb-search risk-preference elicitation task. Participants’ altruism, cooperativeness and nastiness are proxied by their behavior in a dictator game, a social-dilemma game, and a joy-of-destruction game, respectively. Our experimental procedures follow the best practices for stress-related research in medical science and psychology. In particular, we use the TSST for groups (TSST-G) to induce acute stress in the participants of our treatment group and control the effectiveness of our stressor by measuring participants’ salivary cortisol responses and subjective well-being.

Our theoretical benchmark, the standard economic model of human decision-making relies on the assumption of rationality, according to which agents act to achieve the best achievable outcome given their preferences. This, in interactive situations with other rational decision-makers, might require strategizing, that is forming and acting upon beliefs regarding others’ behavior. Although experimental and behavioral economics have significantly enriched the standard model over the past 4-5 decades, its basic structure remains intact [18]. Given our goal of investigating the impact of acute stress on economic behavior through experimentation in the laboratory, we have chosen a small yet sufficiently diverse set of tasks to characterize our subjects in a way that is relevant for economic theory. Our challenge was to balance time, due to possibly changing levels of acute stress, and simplicity, due to cognitive constraints. Regarding individual preferences, our reference points are the decision-makers’ attitude towards risk and their degree of selfishness. In other words, we measure how willing or unwilling people are to make risky decisions (referred to as “risk attitudes” by economists). Also, how much of their private earnings they are willing to sacrifice to help (“altruism”) or to hurt (“nastiness”) others, and whether they are willing to act against their own monetary incentives to contribute towards a collective goal (“cooperativeness”). As for rationality, that is acting upon one’s preferences, we control for subjects’ willingness to engage in reasoning both in an individual setting (“cognitive reasoning”) and in an interactive setting (“strategic sophistication”).

Clearly, our list of tasks to proxy economic behavior is far from being exhaustive. Our research method is similar in spirit to the one adopted by Lane [17] for his literature review on happiness which focuses on a few “types” of economic behavior (namely, interpersonal behavior through selfishness, trust and reciprocity, in addition to individual behavior through risk and time preferences). Also to the one by Falk et al. [19] who carried out a large survey around the world to measure economic preferences characterized by six measures (risk and time preferences, positive and negative reciprocity, and altruism and trust). For our study, we have selected six reference points to describe individual decision-making inspired by economic theory.

Note that the dominant (standard) economic model of human decision-making lacks reference to any kind of stress and other modifiers of subjective well-being. Our research reflects on the appropriateness of these implicit assumptions, and for that reason it essentially follows a path, paved by economic theory under the influence of behavioral and experimental economics, that might look unusual or even mistaken to numerous social scientists. In any case, we are looking for a bridge. We believe that our research questions fit well into the literature on the effects of acute stress on human behavior surveyed above. For an alternative approach and an alternative model used in neuropsychological research in particular, we refer to the survey by Starcke and Brand [20].

We have striven to carefully follow the experimental methods that have become standard in the empirical analyses of stress in order to deliver observations comparable to those presented elsewhere. Our observations stem from a sample whose size of 192 volunteers exceeds the typical sample size reported by earlier studies in the literature and increases the statistical power of our experiment. Thus, the key strength of our study is that we examine a variety of standard problems that have been extensively analyzed by behavioral economists in the experimental laboratory, while placing the subjects under stress using a rigorous and well-tested method. Past studies have focused on a single decision problem or specific personal characteristics (such as risk attitude), and thus offer only a partial understanding of how acute stress affects human behavior. As a result, our data, collected through an experiment based on a between-subject design (when it comes to acute stress), offer a richer view of our participants’ economic behavior.

Our experimental design relies on the group version [21] of the so-called “Trier Social Stress Test” [22] which constitutes the gold standard for inducing acute psychological stress in the laboratory. Von Dawans et al. [21] validate the “TSST for Groups” in a laboratory setting by reporting a more than three-fold increase in participants’ cortisol levels and a significant increase in their heart rates. The protocol also significantly increased the subjects’ self-reported subjective measures of stress and anxiety. It consists of two tasks (a speech and an arithmetic exercise) to be performed in front of cameras and a committee instructed to withhold any type of feedback except for giving new instructions when a mistake is detected. For a survey on TSST, its history of changes and uses, refer to the one by Allen et al. [23]. Also, Liu et al. [24] report results from a meta-analysis (focusing on gender differences in cortisol response) on TSST and show its reliability. Note that we do not simply take the earlier results at face value, but also measure and control for changes in our participants’ salivary cortisol levels. Section 2 offers more details on our experimental procedures.

We find no empirical support for any short-term change in behavior directly caused by acute stress. Our main conclusion, drawn from binary comparisons between the treatment and reference groups, is that acute stress does not have a significant impact on cognitive skills, strategic sophistication, risk attitudes, altruism, cooperativeness, or nastiness. Regression analysis, controlling for individual psycho-social characteristics, corroborates these findings but also suggests that acute stress significantly decreases men’s risk aversion (as measured by the lottery-choice risk-elicitation task). As detailed below, our findings are based on a conservative approach that considers not only statistical significance, but also size effects and takes alternative definitions of the treatment group (i.e., TSST vs. cortisol response) into consideration.

## 2 Experimental design

Our sessions took place at the experimental laboratory of the Faculty of Political Science and Economics at Waseda University, in Tokyo (Japan), between December 2018 and April 2019. Participants were recruited through online advertisements on the school’s website, which contained an invitation to a monetarily incentivized experiment on decision-making that required no preparation or prior knowledge. Participants had to be native speakers of Japanese and full-time students of Waseda University. When they signed up to participate in our experiment, they were asked to report preexisting medical conditions, any medications that they took on a regular basis, body weight and height, and recent sleeping schedule, along with their gender and age. Given that we controlled stress by measuring cortisol concentration in participants’ saliva, we followed standard practice and excluded applicants based on a number of health-related criteria. For example, Petrowski et al. [25] report statistical results from laboratory conditions in which patients with panic disorder produce no cortisol response to psychosocial stress.

Those who were pregnant, who had mental disorders, who had arrhythmia (irregular pulse) or periodontal disease, those who could not keep the same position for a prolonged time due to a physical condition, or who were taking any medication for a treatment purpose were not selected for our study. We asked the participants not to consume any alcohol on the day of or the day before the experiment. They were asked not to eat, exercise, or take caffeinated drinks one hour prior to the experiment. We sent an email reminder about these restrictions on the day before the experiment. In addition, they were not allowed to drink anything during the experiment. Apart from applying these criteria, we also have filtered the pool of applicants in order to balance the gender ratio of our participant pool.

A total of 194 volunteers showed up for the experiment and 192 of them completed it. One participant abandoned the experiment shortly after the tasks required as part of the TSST were announced and described, and another participant did so upon arrival to the computer room (before the computerized tasks were started) complaining about headache. They received the show-up fee and were excluded from the analysis given that they did not participate in the decision-making part of the experiment. Overall, our results are based on data collected from 192 participants. Some of the statistical estimates however work with less than 192 observations, because a few saliva samples turned out to be unusable and one light bulb malfunctioned at a computer booth in one session. Note that all our tables that report statistical results specify the number of observations for each estimate.

Participants were not allowed to participate in more than one of our experimental sessions. The study was approved by the Ethics Review Committee on Human Research of Waseda University under approval case number 2017-016(1).

### 2.1 Experimental procedures

Participants were invited to a classroom near the experimental laboratory. All sessions took place in the afternoon, between 12:45 and 17:05, in a sequence of three sections. Each section lasted for about two hours. Upon arrival, participants were informed about important procedural matters (particularly the functioning of the tool kit for taking saliva samples), and then they filled out a short questionnaire on their sleeping schedule on the day of the experiment, illness over the last three months, the medication they took, their alcohol consumption on the previous day, and the time at which they had lunch, as well as what they had for lunch. They were given an ID number, and they were told that the experiment was of a scientific nature, that the data were all recorded anonymously, and that payment would be made privately in cash at the end of the session. Participants were requested to follow the instructions given by the experimenters and not to talk to each other. They signed a consent form explaining their right to abandon the experiment at any moment in time.

Each experimental session had a maximum of 10 participants, randomly sorted into two groups of similar size. The maximum number of subjects in a group was five and the minimum was four. Participants in the same group moved together during the experiment following the protocol described below.

In the TSST treatment, groups were guided into a quiet classroom (near the classroom used for reception and general instructions but not visible from the control group’s room) in which a committee with two members (wearing white lab coats and showing neutral faces) and clearly visible video cameras were waiting for them. First each participant was requested to deliver a two-minute speech (summarizing past achievements and skills for a mock job interview) in front of the committee, the video cameras, and their peers after two minutes of preparation, and then to perform some mental arithmetic exercises for 80 seconds. The arithmetic exercise consisted of serial subtraction in front of the committee and peers. Participants were required to subtract 17 from a randomly assigned four-digit number and keep subtracting for 80 seconds. When an arithmetic mistake was detected, the committee instructed the participant to start over. They were told that the quality of their speech would be evaluated by a professor at Waseda University later on, that the mental arithmetic task was easy enough for elementary students, and also that their performance would be recorded. Each participant performed these tasks standing up, while others (including the committee members) were seated. The order of performance was random so that participants could not anticipate their turn. The TSST consists of the above-described tasks. It is a frequently used, reliable for inducing stress in human participants in the experimental laboratory [22]. As detailed above, we used its more time- and cost-efficient group version, the “Trier Social Stress Test for Groups” (TSST-G) introduced by von Dawans et al. [21], which allows for subsequent interaction among participants under acute psychological stress. For simplicity, we use the “TSST” and “no-TSST” labels when referring to our treatments.

Individuals assigned to the no-TSST treatment group remained in the initial classroom and were allowed to read quietly. They were not allowed to sleep, talk to each other, or use any electronic devices.

Upon completion of the TSST or a 40-minute waiting time (in the no-TSST treatment), groups were led into the computer rooms (experimental laboratory) by an assistant and were seated at computer terminals separated by walls from each other. We used two adjacent computer rooms (3-803 and 3-804 on the Waseda main campus) which allowed us to work with two groups of up to five participants with five minutes of delay between them. We carried out three pairs of parallel sessions each day in a sequence. The computer rooms were dark, and each booth had a lightbulb installed emitting white, blue, or red light. This choice of colors was inspired by studies reporting (potentially beneficial) effects of blue light on performance, general mood, and stress levels (e.g., [26, 27]). White constitutes a *natural* reference point, while red was chosen for control as another *unnatural* light color. The possible effect of light color is not of primary interest here, but we carefully controlled for it in our statistical analysis. All our sessions took place in the afternoon during the Japanese winter season in two computer laboratories with closed curtains to reduce light contamination from the outside. All bulbs in the same room were of the same color. Participants were asked to read and follow instructions received on-screen. All decisions were made and all interactions occurred through computers.

The first computerized task consisted of a simple reaction-time test implemented in PsychoPy [28]. The screen displayed a single letter (Z or Y) for 0.5 seconds in a random sequence of 60 items, and participants were required to hit a certain key (J) on the keyboard when a specific letter (Z or Y) appeared, in a maximum of one second after the stimulus, and not to hit any key when the other letter was displayed. Around half of our participants had to respond to Z’s and the other half had to respond to Y’s. This task lasted for five minutes and was not incentivized monetarily. We do not use the data recorded during this task; they will be discussed elsewhere.

The second computerized task lasted for 15 minutes and consisted of a sequence of situations in which participants were asked to make decisions for monetary gains. This part was implemented in z-Tree [29] and included the following individual and multiple-person problems:

- Cognitive Reflection Test (CRT) with three questions,
- two-person dictator game (with an endowment of ¥1000),
- five-person beauty-contest game (for a ¥500 prize),
- tasks eliciting risk attitude (an open question, three lottery-choice questions, the bomb-search risk-preference elicitation task).
- two-person social-dilemma game,
- two-person joy-of-destruction game (with a maximum harm of ¥1000).

S5 Appendix presents the original zTree screens and their English translation. All participants went through the same sequence of problems in the order as listed above. They were informed about the outcome of each problem before moving to the next one. We decided not to randomize the order of problems for two reasons. Firstly, our experimental sessions were carefully timed to ensure that tasks, questions and requests for saliva samples happen exactly at the same moment (with the administration of the stressor as a reference point) across all our sessions. Secondly, as some of the tasks involved interaction with other participants, everyone in the same session had to enter each stage in a coordinated fashion. Given that we kept the tasks relatively diverse and their number small, it is unlikely that participants experienced cognitive fatigue or could learn to make “better” decisions as moving along the sequence of tasks. More importantly, our observations for estimating the impact of acute stress on decision-making stem from a between-subject experimental design that does not involve repetition. All the relevant conclusions are based on comparing behavior in the treatment (stressed) group to behavior in the reference (not stressed) group while controlling for as many covariates as possible.

After the second task, the classroom’s lights were turned on, and participants received a short debriefing about the experiment, focusing on the nature and the importance of the TSST for our research. We apologized for the stress induced. S5 Appendix presents the text read aloud to participants as an apology. In the no-TSST treatments, the debriefing did not refer to stress but simply expressed our gratitude for their participation in the experiment.

Finally, the third computerized task lasted for 10 minutes and consisted of a long questionnaire (implemented in z-Tree) with questions targeting personality traits (BFI, PVQ) and personal well-being (CES-D for depression, STAI-T for anxiety proneness), as well as a few philosophical thought experiments on distributive justice. We use a shortened 10-item version of the Big Five Inventory (BFI) [30], the Portrait Values Questionnaire (PVQ) [31], the Center for Epidemiological Studies-Depression (CES-D) [32], and the T-anxiety scale (STAI-T) of the State-Trait Anxiety Inventory (STAI) [33]. These questionnaires are all standard, validated tools that are widely used by psychologists. None of the questions in this task was incentivized monetarily. We use the responses to the BFI, PVQ, CES-D, and STAI-T as controls for personality traits in the statistical analysis reported below but do not work with the items from the philosophical thought experiments; these will be analyzed and discussed elsewhere.

The participants were paid their earnings from the second task together with a ¥500 show-up fee in private at their computer terminal right after the third computerized task and were then allowed to leave. The average monetary earnings, excluding the show-up fee, was around ¥2200. The monetary compensation the participants received was significantly higher than the typical earnings from economics experiments on decision-making in the laboratory at Waseda. According to the official exchange rate at the time of our experimental sessions, it was equivalent to 20 USD and would be enough to buy four lunch boxes on campus. The earnings were also more volatile than usual, with a minimum of ¥200 and a maximum of ¥4000.

We measured participants’ stress levels through saliva samples and simple paper-based questionnaires administered four times during the experiment, as described below. Groups were moved through the experimental phases and tasks with a stop-watch to make sure that the samples were taken in a controlled, timely manner. Recall that all our sessions took place in the afternoon (between 12:45 and 17:05 in a sequence with three sections) during the Japanese winter season in order to control light conditions; this also helped us control for the diurnal cycles of cortisol secretion.

1. After arrival, as part of the general instructions, participants were shown and practiced how to use the saliva sample kits. A *test* sample was collected as they did so, but its results are not used in the statistical analysis. The transcript of these instructions is presented in S5 Appendix.
2. Five minutes before the TSST, after the two-minute speech-preparation time. This is the benchmark measurement (minute -5). Participants in the no-TSST treatment supplied an initial sample after receiving general instructions, matching the schedule of the TSST treatment. Note that the TSST is our minute 0 reference.
3. Twenty minutes after the first sample, after the TSST or after the waiting time in the no-TSST treatment.
4. Thirty minutes after the first sample, once groups moved to the computer rooms and before the computerized tasks.
5. Fifty-five minutes after the first sample, after the second computerized task and before debriefing (and the third computerized task with questionnaires).

Our experimental design includes four saliva samples, out of which the first serves as the reference point. The second and the third were meant to capture the peak in salivary cortisol concentration that typically occurs 20-30 minutes after the stressor [7, 21], while the fourth was timed to show its decay. These features of the experimental design are in line with the guideline set by Shields [34] for studies exploring stress and cognition. While a larger number of test could have offered a more accurate picture of the evolution of cortisol (and acute stress), its costs in terms of a smaller number of observations (due to our hard budget constraint) and repeated interruptions in the flow of the experimental session would probably have outweighed its benefits. Also, providing enough saliva for clinical tests more than four times could have been physically too demanding for quite a few of our participants.

**Fig 1:**
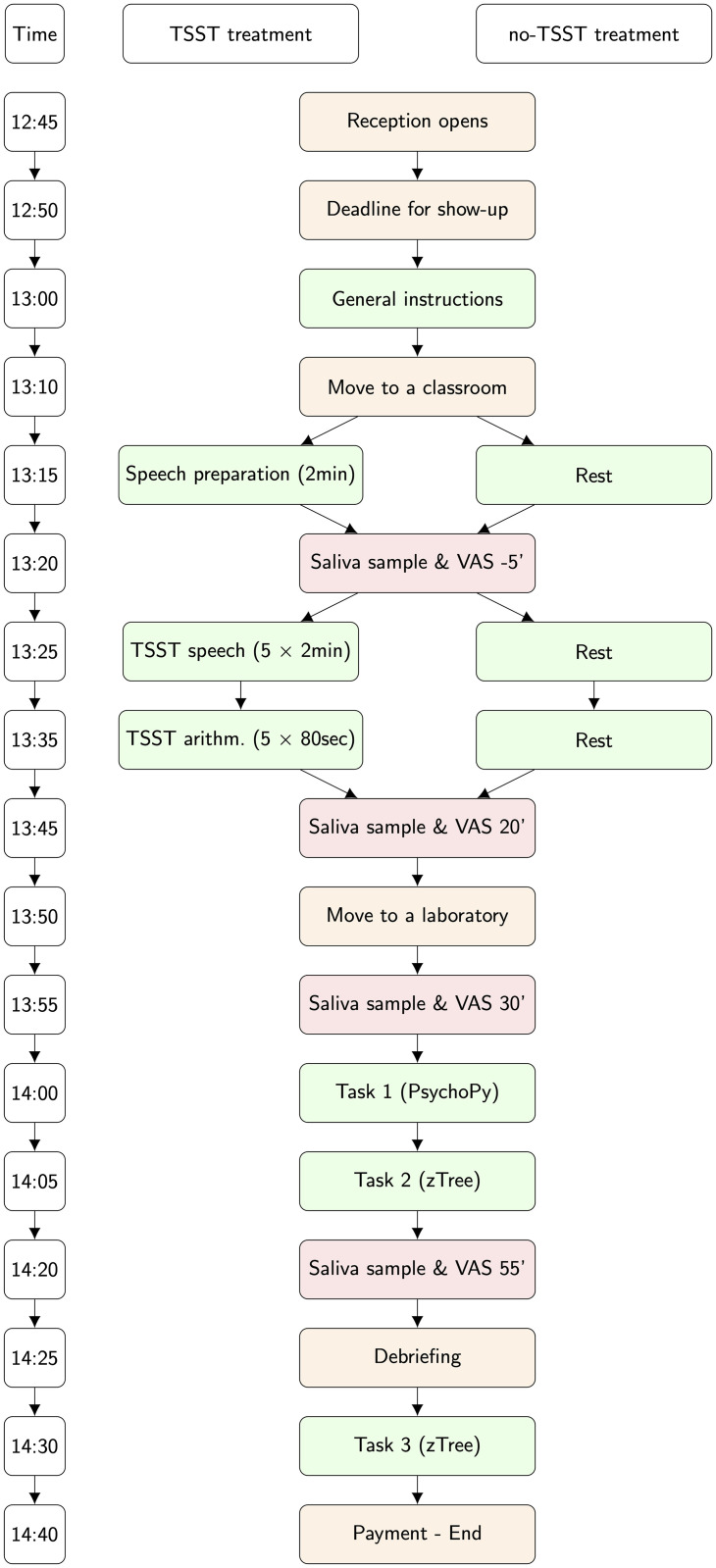
Timing of a typical session in section 1 with or without TSST.

Along with providing saliva samples, participants were also requested to self-report their well-being. Together with each saliva sampling toolkit, participants were given a sheet of paper with three printed 10 cm-long horizontal visual analog scale (VAS) representing a 0-to-100 scale (ranging from 0 to 100), on which participants were asked to provide ticks in answer to questions on their current stress, excitement and fatigue levels, respectively. After each saliva sampling, participants were asked to answer the following three questions on VAS: “How much stress do you feel?” “How excited are you?” “How tired are you?” Fig 1 offers a visual summary of the timing of our experimental sessions.

### 2.2 Outcome variables

We have chosen a limited number of simple decisions problems with which to analyze participants’ behavior under stress. The problems have all appeared and received extensive attention in the experimental and behavioral economics literature. The list below offers a brief description of each one and offers a reference to the standard economic model of decision-making that serves as our benchmark. Note that we refer to the tasks and the related behavioral concepts as customary in economics, in particular in game theory and behavioral economics. S5 Appendix presents the original Japanese zTree screens (and an English translation beneath each one) used for the monetarily incentivized tasks.

- Cognitive Reflection Test [35] with the three standard questions (about the baseball bat, the water lily, and the machines), used to measure participants’ tendency to override a quick but typically incorrect response and engage in reflection to find the correct answer. Questions appeared on the screen one by one, answers had to be entered in within 20 seconds and each correct answer was rewarded with ¥100. The related statistical variable counts the number of correct answers given in the 20 seconds/question timeframe. It proxies the decision-maker’s reasoning capacity, a key—albeit hidden—element in the economic model of rational decision-making which presupposes careful considerations and limitless cognitive abilities. This test has been extensively used in empirical research [36], and it has been shown to outperform other measures (e.g., the Raven test [37]).
- A two-person dictator game in which each participant had to decide how much money to keep from an exogenously assigned endowment of ¥1000 and how much to give to a partner randomly and anonymously selected and assigned from the group of participants. The amount given to the partner in the dictator game is typically interpreted as a numerical measure of altruism [38], because a decision-maker with standard self-regarding preferences would keep the entire endowment. Our variable records the amount of money kept by the dictator. Participants were required to make a decision in a maximum of three minutes. Pairs were formed in such a way that each participant acted as a dictator and as a partner (or receiver) exactly once.
- The beauty-contest game is a guessing game in which each participant simultaneously chooses a number between 0 and 100 (limits also permitted). We implemented the version in which the participant whose number lies the closest to $\frac{2}{3}$ of the average of all chosen numbers wins a fixed prize of ¥500, while all other participants receive nothing. Most groups had five participants (some had 4). This game is dominance solvable, but a large number of iterations are required due to the large number of available strategies. The unique Nash equilibrium of the game (independently of group size) is when each participant chooses the number 0. Experimental and behavioral economists interpret the chosen number as a measure of strategic sophistication, given that larger numbers correspond to dominated strategies and indicate naïve play and smaller ones indicate higher levels of iterative thinking about others’ behavior in order to maximize one’s expected gain [39]. Participants were required to make a decision within three minutes.
- Risk attitudes play a key role in describing decision-making under risk in economic models. They describe how willing or unwilling the decision-maker is to take risk. The literature does not agree on the best method of eliciting risk attitudes, so we have applied three different approaches.
  - Following Dohmen et al. [40], we requested participants to answer the following question on a scale from 0 to 10: “How do you see yourself: are you a person who is generally willing to take risks, or do you try to avoid taking risks?” Larger values for the related variable indicate more willingness to take risk. Participants were required to give an answer in a maximum of one minute.
  - Inspired by Falk et al. [19], we also offered three monetarily incentivized choices between a fixed lottery (which paid ¥50 with 50% probability and ¥650 with 50% probability) and varying sure payments (¥350, ¥300, and ¥250). Participants who choose the lottery more often (recorded as our variable) are more willing to take risk. Participants were required to make their choices within three minutes.
  - We also implemented the bomb-search risk-elicitation task [41] in which participants had to decide how many to open of the 100 boxes that appeared on the screen, one of which contained a “bomb”. Participants received ¥1 for each box they opened if the one with the bomb remained unopened and nothing otherwise. Choosing a larger number of boxes indicates more willingness to take risk. Participants were required to make a decision within three minutes.
- Participants’ cooperativeness was measured in an extreme social-dilemma situation in which they were randomly assigned into pairs and had to decide simultaneously whether to keep and pocket ¥250 or send it to their partner, in which case the money was doubled. This game is an instance of the well-known “prisoner’s dilemma”, albeit without Tucker’s original story [42], in which personal and group interests are opposed. The game played by agents with standard self-regarding preferences has an equilibrium in dominant strategies in which both agents keep their initial endowment. The equilibrium outcome however is not efficient (not socially optimal), as agents forgo the additional earnings that could have arisen from the doubling of the monetary amounts had the money been sent to the opponent. We will refer to this game as a “gift-exchange game”, even though the experimental-economics literature uses that name to refer to a more complex two-person interaction [43]. Sending one’s endowment (i.e., a gift) to one’s partner is going to be interpreted as cooperative behavior. The related variable takes a value of 1 if the participant decides to cooperate and 0 otherwise. Participants were required to make a decision within three minutes. Pairs were formed in such a way that each participant acted as a sender and as a receiver exactly once.
- Finally, our list includes a measure of nastiness (the dark side of human nature) through a game called the “joy of destruction” [44]. We offered each participant the opportunity to destroy no more than ¥1000 of another participant’s earnings. They were shown the accumulated monetary gains of a randomly chosen participant in an anonymous manner and were allowed to destroy (at no cost and at no gain) at most ¥1000 yen of it. This game is the negative counterpart of the dictator game, as the typical economic decision-maker with self-regarding preferences would not want to incur in costs that bring no personal benefits in monetary term. The amount destroyed is therefore interpreted as a numerical measure for one’s “nastiness”. Participants were required to make a decision within three minutes. Pairs were formed in such a way that each participant acted as an active and as a passive party exactly once.

The above list is far from being an exhaustive one of the behavioral traits and patterns in which experimental and behavior economists have shown interest over the past five decades. Beside the cited references above, we refer our readers to the two volumes of the Handbook of Experimental Economics [45, 46] which contain ample discussion on the dictator game (chapter 4 in volume 1, chapter 4 in volume 2), the beauty-contest game (chapters 3 and 7 in volume 2), risk attitudes (chapter 1 in volume 1), and social dilemmas (chapter 2 in volume 1).

Overall, we have tried to assemble a basic set of decision-problems that have played a prominent role in the literature and that are easy to explain relatively quickly. Recall that stress levels decay with time and that our cortisol concentration measurements imposed strict time limits and a strict schedule. As detailed in the introduction, our goal was to choose a sufficiently diverse set of tasks to be able to characterize participants’ preferences to some extent and to be able to judge whether their decisions are made as carefully and in such a strategic manner as assumed by the standard economic model of human decision-making.

## 3 Results

### 3.1 Stressor validation

**Fig 2:**
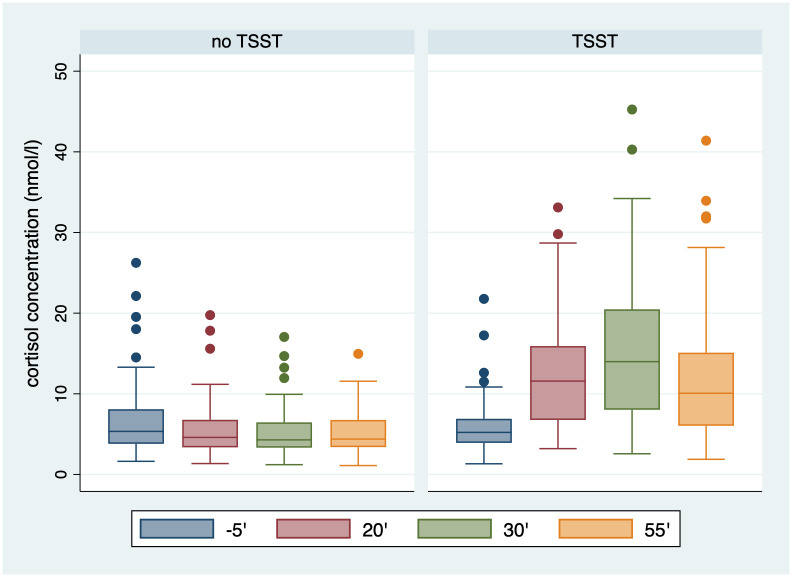
Evolution of cortisol concentration with and without TSST.

As shown in Fig 2, the TSST had a positive impact on participants’ cortisol levels which, on average, peaked in the sample taken 30 minutes after the stressor was administered (30’). Following Cahlíková and Cingl [7], we categorize participants as stressed when they experience a cortisol-level change of more than 2.5 nmol/l anytime during the experiment relative to the sample taken right at the beginning of the experiment (-5’). Recall that, when referring to the timing of saliva samples, we treat the TSST as minute 0.

We summarize the validation of the stressor included in our experimental design in the following result.

**Result 1** *The TSST has resulted in stress (i.e., in a cortisol-concentration response of more than 2.5 nmol/l) in 75% of our participants*.

**Fig 3:**
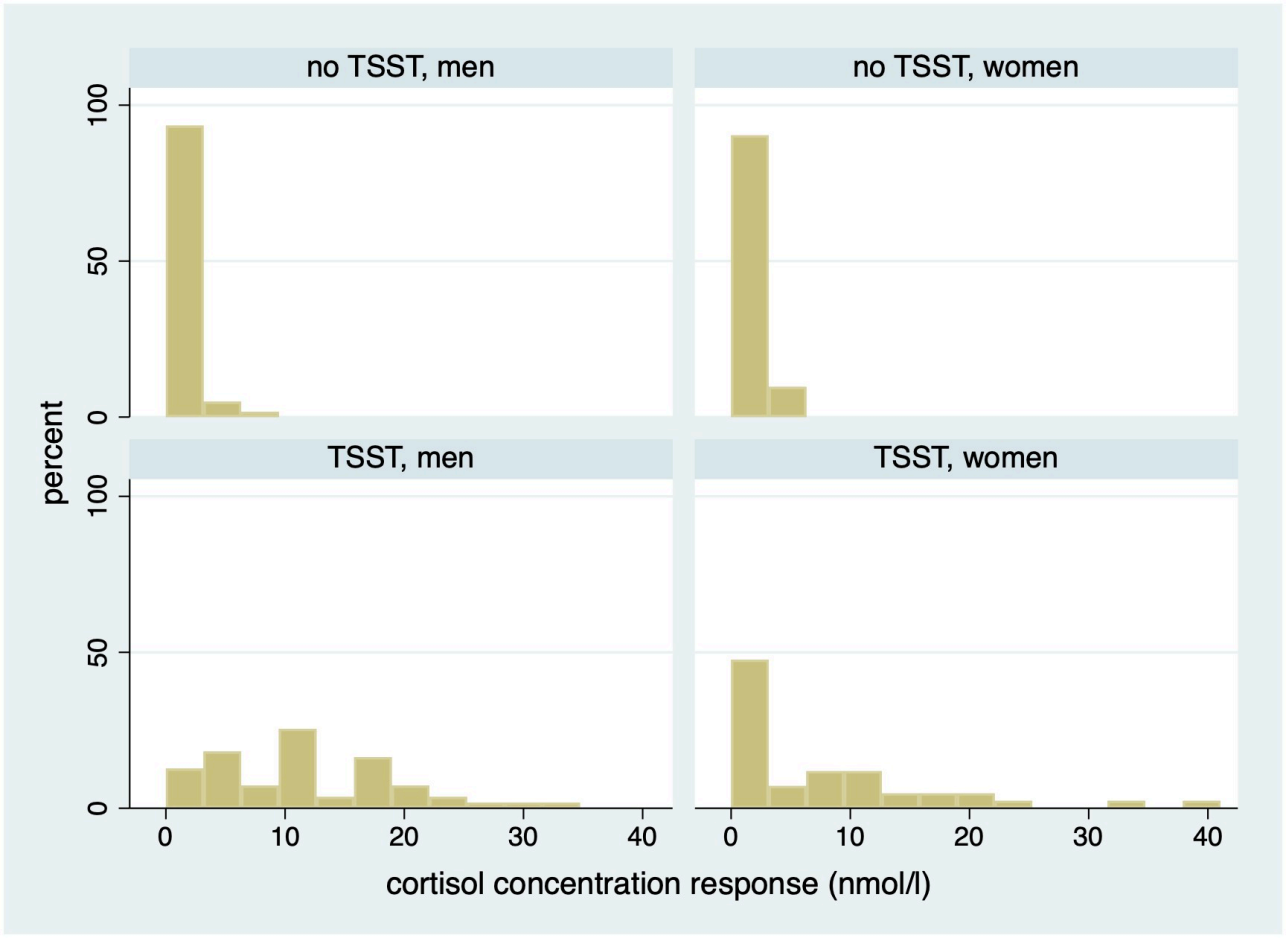
Distribution of cortisol concentration response with and without TSST.

Interestingly, 9% of those participants who did not participate in the TSST also experienced stress during their participation. We attribute that to the experiment itself—to spending a considerable amount of time in a tightly controlled environment, periodically producing saliva samples, and making decisions under time pressure for monetary gains. Fig 3 displays the distribution of peak cortisol concentration response, showing that TSST tends to provoke an increase that varies largely across the participant pool. Our data also reveal that the TSST results in stress significantly more often in men than in women (89% vs. 57% of the cases, respectively; *p*-value = 0.0003 for a large-sample *z*-test of proportions).

The color of the light under which participants were required to make decisions does not seem to have a significant impact on the impact of TSST on cortisol concentration levels. The proportion of participants that were categorized as stressed after the TSST is 78% for white light, 81% for red, and 67% for blue. Although the distribution of cortisol-concentration response levels look slightly more skewed for white light than for the other two (see Figs 1, 2 and 3 in S1 Appendix, pairwise comparisons based on the Kolmogorov-Smirnov equality-of-distributions test reveal no statistically significant differences (combined *p*-value = 0.27 for red and white, combined *p*-value = 0.68 for blue and white, combined *p*-value = 0.17 for red and blue). The parametric pairwise proportion tests deliver the same conclusions, with *p*-value = 0.76 for red and white, 0.30 for blue and white, and 0.18 for red and blue. With a *p*-value = 0.77 in a Spearman’s rank correlation test, we can not reject the hypothesis that color and stress are independent.

Even though the 2.5 nmol/l threshold is often used in the literature to categorize people as stressed, some researchers (e.g., [47]) consider it overly conservative and suggest using a lower limit of 1.5 nmol/l. As a robustness check, we have repeated the above-described analysis with that lower value and find that the qualitative result on the validation of the stressor does not change; only minor numerical changes appear. That is, with a threshold of 1.5 nmol/l for change in cortisol concentration, the TSST results in stress in 80% of our participants, while 15% are categorized as stressed without going through the TSST. The TSST induces stress significantly more often in men than in women (91% vs. 64% of the cases, respectively, *p*-value = 0.0013). The proportion of participants that were categorized as stressed after the TSST is 78% for white light, 90% for red, and 69% for blue. The distance between these percentages suggests that blue light helps the avoidance of stress caused by TSST and red light adds to it. Pairwise proportion tests show that those distances to the benchmark white color are not statistically significant (*p*-value = 0.17 for red vs. white, and *p*-value = 0.44 for blue vs white), while the difference between blue and red is (*p*-value = 0.04). Nevertheless, with a *p*-value = 0.22 in a Spearman’s rank correlation we can not reject the hypothesis that color and stress are independent. Moreover, the Kolgomorov-Smirnov test compares the cortisol-distributions across subsamples defined by color. We conclude that the color of light has no significant impact on the effect of TSST.

**Fig 4:**
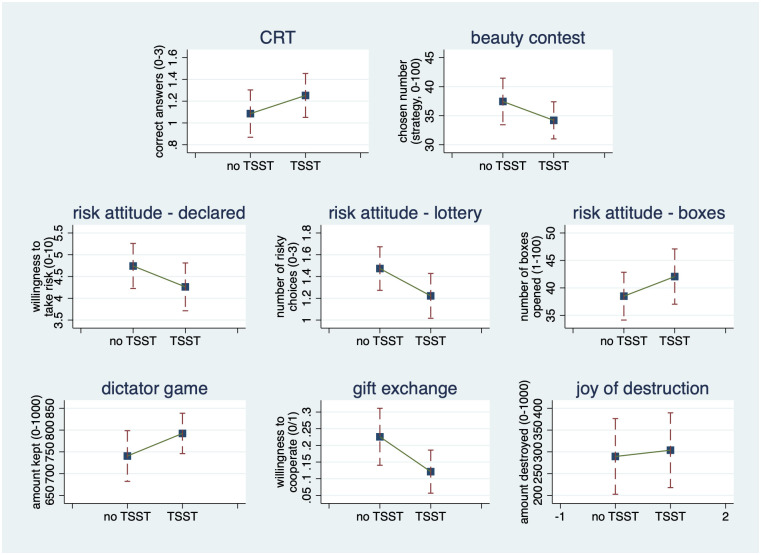
The effect of acute stress on decision-making. TSST treatment effects visualized by point estimates and 95% confidence intervals.

**Fig 5:**
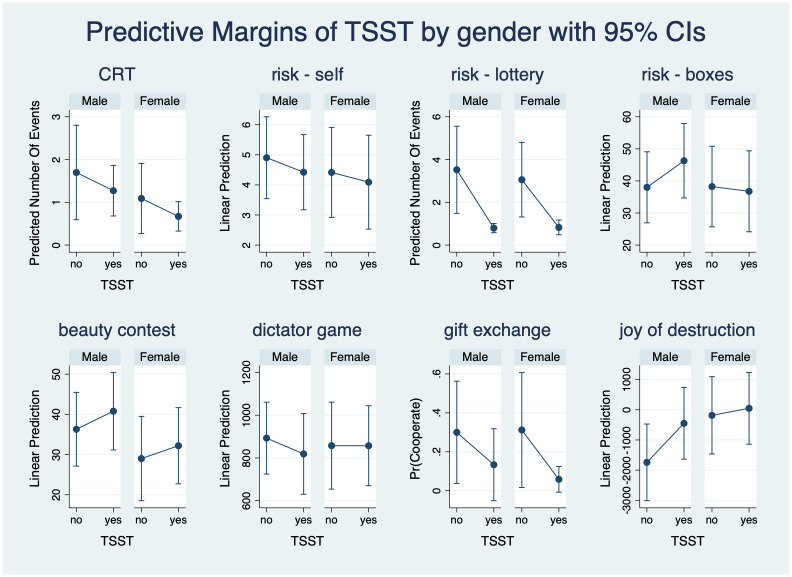
Predictive margins of TSST by gender with 95% CIs.

Regarding participants’ self-declared wellness levels measured on visual analog scales (VAS), the TSST had a significant impact on stress, excitement, and fatigue, but with different time patterns. Figs 4, 5, and 6 in S2 Appendix show the evolution of self-reported wellbeing (i.e., stress, excitement, and fatigue, respectively) in a box-plot form similar to that shown in Fig 3. The description that follows relies on binary comparisons between the TSST treatment group and the reference group via the Wilcoxon rank-sum test (or Mann-Whitney two-sample test for independent samples) reported in Table 1 in S2 Appendix. Participants who went through the TSST arrived significantly more stressed to the experimental laboratory and remained so until the last measurement (at 55’), when statistical differences vanished. In terms of excitement (or arousal), the TSST treatment group shows a similar pattern: Self-reported excitement levels significantly exceed those of the benchmark group at the beginning (for at least 20 minutes), after which they become statistically indistinguishable. Interestingly, for excitement, this equality happens around the third measurement (at 30’), after which the benchmark group reports higher levels than the TSST group. Differences in fatigue across the two groups are less pronounced but are significantly different for the first three measurements, with the TSST group reporting higher levels.

### 3.2 Decisions under acute stress

We now turn our attention to the impact of stress on behavior as typically measured through a number of standard problems and interactive games in the economic experimental laboratory. Table 1 reports the mean values for all our variables of interest, along with effect sizes measured through Cohen’s d, *p*-values for the Wilcoxon rank-sum test (also known as the “Mann-Whitney two-sample test”) for most of them, and *p*-values for a large-sample *z*-test in case of proportions. We make comparisons for the subsample of both the participants who participated in the TSST (top panel of the table) and the participants who were categorized as stressed during the experiment according to one or the other threshold for change in cortisol level (bottom two panels of the table). Our main finding is summarized in the following result.

**Table 1:** Stress and changes in behavior (binary comparisons and treatment effects).

	CRT	beauty contest	declared	risk attitude lottery	boxes	dictator game	gift exchange	joy of destruction
no TSST	1.086	37.45	4.742	1.473	38.49	740.4	0.226	289.4
obs.	93	93	93	93	93	93	93	93
TSST	1.253	34.20	4.263	1.222	42.06	792.3	0.121	303.8
obs.	99	99	99	99	99	99	99	99
*p*-value	0.240	0.311	0.172	0.0847	0.417	0.293	0.0549	0.858
Cohen’s d	-0.159	0.180	0.179	0.247	-0.151	-0.199	0.278	-0.0333
not stressed	1.083	38.33	4.602	1.407	39.27	754.3	0.204	296.2
obs.	108	108	108	108	108	108	108	108
stressed (2.5+)	1.309	31.99	4.432	1.296	42.12	788.3	0.136	296.4
obs.	81	81	81	81	81	81	81	81
*p*-value	0.123	0.0568	0.633	0.514	0.609	0.676	0.224	0.921
Cohen’s d	-0.216	0.356	0.0639	0.109	-0.121	-0.130	0.179	-0.000444
not stressed	1.102	37.79	4.684	1.398	39.12	762.9	0.204	285.4
obs.	98	98	98	98	98	98	98	98
stressed (1.5+)	1.264	33.27	4.363	1.319	41.97	775.3	0.143	308.0
obs.	91	91	91	91	91	91	91	91
*p*-value	0.263	0.227	0.392	0.650	0.689	0.929	0.268	0.654
Cohen’s d	-0.155	0.251	0.121	0.0776	-0.120	-0.0472	0.161	-0.0524

Note: *p*-values for a large-sample *z*-test of proportions (for gift exchange) and the Wilcoxon rank-sum test (for all other).

**Result 2** *Acute stress does not have a significant impact on cognitive skills, strategic sophistication, risk attitudes, altruism, cooperativeness, or nastiness*.

The qualifier “significant” refers here to both statistical significance at the usual levels and effect size. Hence, Result 1 is based on a conservative approach that also takes alternative definitions (i.e., TSST vs. cortisol response) of the treatment group into consideration.

Fig 4 offers a visual summary of Result 1 by displaying the TSST treatment effects with the help of point estimates and symmetric 95% confidence intervals around them.

Given our sample sizes and *α* = 0.05, the statistical power of the applied two-tailed Wilcoxon rank-sum test to detect a medium effect size (i.e., when Cohen’s *d* = 0.5) is 0.89 for the split according to TSST (with subsamples of 91 and 99 observations) and *stressed (1.5+)* (with subsamples of 98 and 91 observations) and 0.88 for *stressed (2.5+)* (with subsamples of 108 and 81 observations). These numbers decrease to 0.48 and 0.47 when the target effect size is *d* = 0.3, and further to 0.25 and 0.24 when it is *d* = 0.2. We have carried out the power analysis with the help of G*Power [48]. It is important to note that these estimates for statistical power are for a single (isolated and independent) statistical test. Our results however are related to a survey-type set of questions and tasks that stem from the same respondent. While our measurements capture sufficiently different aspects of human decision-making (except for the three methods targeting risk preferences) and none of the questions was repeated, some might argue that the statistical tests that we perform are not fully independent. Although our main comparisons are across treatments (related to stress) and across participants (based on a between-subject design), the above-reported estimates are to be interpreted as upper bounds for statistical power.

When looking at the change caused by TSST or stress in average behavior, we recorded an increase in the number of questions answered correctly in the cognitive reflection test and a decrease in the number chosen in the beauty-contest game (i.e., an increase in strategic sophistication). Note that our between-subject design does not allow for looking at changes in individual behavior. It seems as if participants paid more attention to their tasks and made somewhat sharper decisions under stress. However both of these effects are small (as measured by Cohen’s d), and none is statistically significant at the usual levels. Following Cohen’s guidelines, we interpret a *d* with an absolute value of around 0.2 as a “small,” one around 0.5 as a “medium,” and one around 0.8 as a “large” size effect [49, p. 25-26]. In the end, our results are in line with the early finding on strategic sophistication under acute stress reported by Leder, Häusser and Mojzisch [1], as we detect no statistically significant differences between the treatment and benchmark groups in terms of the number chosen in the (first round of a) beauty-contest game. They do differ from the conclusion reached by Bendahan et al. [4] that acute stress increases decision-makers’ willingness to take risks.

To measure risk attitudes, we have used two monetarily-incentivized tasks and a self-evaluative open question. When answering the latter, post-TSST participants and those who were stressed claimed that they were somewhat less keen on taking risks in their life. In line with this claim, they also chose the safe payment more often against the risky lottery. At the same time, however, they decided to open a few more boxes in the bomb elicitation task, a behavior that signals more willingness to take risks. Except for the difference in lottery choices between the TSST group and its benchmark, none of the listed changes is large or even statistically significant at the usual levels. Our conclusions on risk-attitudes being unaffected by acute stress are in line with the results reported by Sokol-Hessner et al. [3].

In terms of altruism (money sent in the dictator game), cooperativeness (money sent in the gift-exchange game), and nastiness (money destroyed in the joy-of-destruction game), the TSST and stress on average have resulted in lower altruism, less cooperativeness, and more nastiness. Again, however, none of the listed changes is large or statistically significant at the usual levels, except for the comparison between the TSST group and its benchmark for the gift-exchange game.

### 3.3 Robustness check: Redefined treatment group

When considering cortisol levels, instead of having participated in TSST as a proxy for stress, none of the treatment effects is statistically significant, except for a smaller number of boxes opened (hinting at more risk-aversion) when the 2.5 nmol/l threshold is used for assigning participants into the *stressed* category.

Inspired by the literature on gender differences in behavior under acute stress, we also performed separate binary comparisons between the treatment groups and the corresponding benchmarks for the two self-reported gender groups. Table 2 in S3 Appendix presents all the numerical details. The results suggest that stress increases cognitive ability in men and reduces their cooperativeness (both effects are small). The only other statistically significant effect observed is that women choose smaller numbers in the beauty-contest games (hinting at increased cognitive ability) but only when cortisol response with the 2.5 nmol/l threshold is used to define the treatment category.

### 3.4 Robustness check: Regression analysis

We perform a robustness test for the above results by estimating the impact of the TSST and stress while controlling for a large number of variables. The controls include

- demographic variables (i.e., gender, age, height and weight, college grade, college major),
- experimental-session characteristics (i.e., date, laboratory ID, section ID, light color),
- a measure of cognitive skills (via the CRT), and
- measures for personality traits (BFI and PVQ), and
- personal well-being (CES-D and STAI-T).

S4 Appendix describes the applied scoring rules and presents a descriptive analysis of the control variables in Tables 3, 4, and 5 in S4 Appendix. It is important to point out that our reference, the standard economic model of individual decision-making, is blind to gender, age, weight, and all the other factors listed above. We nevertheless include them in the regression analysis in order to control for possible unduly-ignored effects while our main interest remains in exploring the impact of acute stress. The use these control variables has numerous precedents in the literature both in economics and medicine in spite of not appearing in the reference model of human decision-making (e.g., gender in [12, 15, 36], cognitive skills in [37], personality traits in [7, 50], personal well-being and psychiatric disorders in [51]).

Given that we observed significantly different reactions to the stressor according to the participants’ gender, our regressions include the gender and the treatment variables as an interaction terms as well. The estimation results displayed in Table 2 confirm our null result from the above-described binary comparisons, with a few exceptions summarized in the following result. The complete version of Table 2 appears in Tables 6, 7, and 8 in S4 Appendix. Note that the last two rows of the table report *p*-values for hypothesis tests related to treatment effects for male and female participants separately.

**Table 2:** Stress (TSST) and changes in behavior (regression analysis).

	CRT	beauty contest	declared	risk attitude lottery	boxes	dictator game	gift exchange	joy of destruction
TSST	-0.289	4.493	-0.482	-1.488***	8.280	-73.69	-1.676	1294.7
	(0.531)	(8.368)	(1.123)	(0.384)	(10.12)	(153.2)	(2.324)	(998.0)
female	-0.443	-7.288	-0.489	-0.141	0.241	-35.15	0.1000	1558.5**
	(0.280)	(5.319)	(0.824)	(0.243)	(6.458)	(111.8)	(0.970)	(720.7)
TSST × female	-0.197	-1.300	0.157	0.179	-9.774	73.31	-1.408	-1062.7
	(0.294)	(5.449)	(0.757)	(0.250)	(8.005)	(112.7)	(1.266)	(785.9)
controls	✓	✓	✓	✓	✓	✓	✓	✓
Observations	191	191	191	191	191	191	189	191
R2			0.371					
Pseudo R2	0.101	0.0489		0.0779	0.0352	0.0387	0.358	0.0916
*p*-value: *β*_*TSST*_ = 0	0.587	0.592	0.669	0.000107	0.414	0.631	0.471	0.197
*p*-value: *β*_*TSST*_ + *β*_*TSST* × *female*_ = 0	0.503	0.812	0.836	0.473	0.224	0.516	0.266	0.178

Note: CRT, risk attitude (lottery): Poisson regression; beauty contest, risk attitude (boxes), dictator game, joy of destruction: TOBIT regression with robust standard errors; risk attitude (declared): OLS with robust standard errors; gift exchange: LOGIT regression with robust standard errors. Estimated coefficients significantly different from zero at

*10%,

**5%, and

***1%.

Controls for demographics, session characteristics, cognitive skills, and personality traits.

**Result 3** *Acute stress has no significant impact on cognitive skills, strategic sophistication, risk attitudes, altruism, cooperativeness, or nastiness, except that it significantly decreases men’s risk aversion (as measured by the lottery-choice risk-elicitation task)*.

Note that the men in the TSST treatment group chose the risky lottery significantly less often (on average, 1.4 less often in the three questions) than did the men in the reference no-TSST group. Otherwise, our regressions find no statistically significant effect on decisions or behavior. This finding seems to be in line with earlier reports on increased risk aversion induced by acute risk in men [6, 7]. Fig 5 plots the treatment effects together with 95% confidence intervals for a quick and visual summary of our results. It is noteworthy that we find no gender effects in the no-TSST group either, except that females destroy significantly more of their partner’s gains in the joy-of-destruction game. The general no-result and the statistically significant gender effect are robust to changes in the measurement of acute stress. As Tables 9 and 13 and Figs 7 and 8 in S4 Appendix show, it does not matter whether we proxy for acute stress using participation in TSST or using a cortisol concentration response of more than 1.5 nmol/l or more than 2.5 nmol/l. As in Table 2 reported in the main text, S4 Appendix contains the full versions of Tables 9 and 13 (Tables 10, 11, and 12, and Tables 14, 15, and 16, in S4 Appendix respectively) and the figures with the estimated treatment effects (Figs 7 and 8 in S4 Appendix). However, we are unable to identify any statistically significant change in risk attitudes when using the other two alternative definitions of stress.

## 4 Conclusions

We have used the TSST for groups to induce acute stress in participants randomly assigned to the treatment group and studied behavior as is usually done in the experimental-economics laboratory. Our main contribution to the literature is a no-result, given that we could not detect any significant impact of acute stress on participants’ cognitive skills, strategic sophistication, risk attitudes, altruism, cooperativeness, or nastiness. The only statistically significant change observed is that stress seems to significantly decrease men’s risk aversion as measured by the lottery-choice risk-elicitation task.

When considered alongside the conclusions reported by the studies described in our literature review, our results are in line with the findings by Leder, Häusser and Mojzisch [1] that behavior is unaffected by stress in the first round of the beauty-contest game. However, we are unable to replicate the results on changes in risk attitudes (pushed towards more risk-aversion) and on more self-oriented behavior under stress shown by Bendahan et al. [4], or the increase in female cooperativeness under stress observed by Youssef et al. [8] and von Dawans et al. [9]. Our results on increasing risk aversion induced by stress in men seem to be more in line with those reported by Kandasamy et al. [6] and Cahlíková and Cingl [7]. Nevertheless, our regression results on risk aversion should be treated with caution, as it is observed for only one specific measurement of risk attitude and one specific way of defining stress (TSST).

In conclusion, acute stress does not invalidate the usual assumptions made by economists regarding decision-makers’ consistency [16], nor does it change the basic behavioral patterns extensively studied and reported by experimental and behavioral economists. In other words, the standard economic model of human decision-making does not need to be amended to account for the presence of acute stress. However, these conclusions must not be interpreted as the final word in the economic analysis of stress, as many open questions remain. Pacák and Palkovits [52] point out that *stress* as a medical and scientific concept was introduced in 1936 by Hans Selye in a letter to *Nature*. They define stress “as a state of threatened homeostasis (physical or perceived threat to homeostasis)”. It is unclear, however, whether the operational definition, that identifies acute stress as a salivary cortisol response or some other physiological reaction often used in experiments accurately captures what social scientists understand stress to be. Refer to Buckert et al. [13] and their references for a short discussion on this matter.

## Legends for the data

The following list provides a brief description of the variables included in our database. Note that in oder to make the dataset completely anonymous, we have removed some of our participants’ personal information (in particular, school, age, height, and weight). For that reason, some of the statistical results reported in the manuscript can not be replicated with this database.

- filename → name of the original datafile created by zTree; zTree started: YYMMDD_HHMM
- room→ location; computer room at the experimental laboratory at Waseda University (building no.3): 803 or 804
- section→ section number; several experimental sessions took place in each room on each day: 1, 2, or 3
- color → color of the lightbulb in the booth: white, red, or blue
- TSST→ indicator for subjects who underwent the Trier Social Stress Test: yes or no
- bat→ answer to cognitive reasoning test (CRT) question no.1; correct answer: 0.05; easy wrong answer: 0.10; no answer: -1
- widgets→ answer to cognitive reasoning test (CRT) question no.2; correct answer: 5; easy wrong answer: 100; no answer: -1
- lake:→ answer to cognitive reasoning test (CRT) question no.3; correct answer: 47; easy wrong answer: 24; no answer: -1
- dictator→ money kept (from a budget of 1000 ¥) in a dictator game: 0–1000
- beauty→ strategy chosen in a beauty-contest game (with the subjects in the same session/room): 0–100; Nash-equilibrium strategy: 0
- risk1→ answer to risk attitude question no.1 (lottery choice): 1 or 2
- risk2→ answer to risk attitude question no.2 (lottery choice): 1 or 2
- risk3→ answer to risk attitude question no.3 (lottery choice): 1 or 2
- risk4→ answer to risk attitude question no.4 (self declared): 0–10
- risk5→ answer to risk attitude question no.5 (bomb task): 1–100
- gift→ strategy chosen in a two-person gift-exchange (prisoners’ dilemma) game: 1 or 2; Nash-equilibrium strategy: 1
- destroy→ strategy chosen in a joy-of-destruction game: 0–1000
- stai1—stai20→ answers to STAI-T for anxiety proneness
- ces1—ces20→ answers to CES-D for depression
- big1—big10→ answers to the Big Five Inventory
- pvq1—pvq40→answers to the Portrait Values Questionnaire
- sex→ respondent’s gender (female or male)
- cortisol0_cal→ salivary cortisol level (nmol/l) during instructions
- cortisol1_cal→ salivary cortisol level (nmol/l) before TSST (-5)
- cortisol2_cal→ salivary cortisol level (nmol/l) 20 minutes after TSST (20)
- cortisol3_cal→ salivary cortisol level (nmol/l) 30 minutes after TSST (30)
- cortisol4_cal→ salivary cortisol level (nmol/l) 55 minutes after TSST (55)
- vas_0_q1, vas_0_q2, vas_0_q3→ answers reported during instructions on the visual analog scale (VAS) to questions on current stress (q1), excitement (q2) and fatigue (q3)
- vas_1_q1, vas_1_q2, vas_1_q3→ answers reported before TSST (-5) on the visual analog scale (VAS) to questions on current stress (q1), excitement (q2) and fatigue (q3)
- vas_2_q1, vas_2_q2, vas_2_q3→ answers reported 20 minutes after TSST (20) on the visual analog scale (VAS) to questions on current stress (q1), excitement (q2) and fatigue (q3)
- vas_3_q1, vas_3_q2, vas_3_q3→ answers reported 30 minutes after TSST (30) on the visual analog scale (VAS) to questions on current stress (q1), excitement (q2) and fatigue (q3)
- vas_4_q1, vas_4_q2, vas_4_q3→ answers reported 55 minutes after TSST (55) on the visual analog scale (VAS) to questions on current stress (q1), excitement (q2) and fatigue (q3)

## Supporting information

S1 Appendix Additional figures and tables related to Stressor validation (TSST and cortisol). (PDF)

S2 Appendix Stressor validation—TSST and self-reported wellness (stress, excitement, fatigue). (PDF)

S3 Appendix Treatment effects by gender. (PDF)

S4 Appendix Regression analysis. (PDF)

S5 Appendix Additional procedural details and instructions. (PDF)

S1 Data (DTA)
