# Supplementary material for: Acute stress does not affect economic behavior in the experimental laboratory
Source: PLoS One. 2021 Jan 7;16(1):e0244881. doi: 10.1371/journal.pone.0244881 (PMC7790397; doi:10.1371/journal.pone.0244881)

S1 Appendix - Additional figures and tables related to Stressor validation (TSST and cortisol)

Fig 1. Distribution of cortisol concentration response with and without TSST by light color.

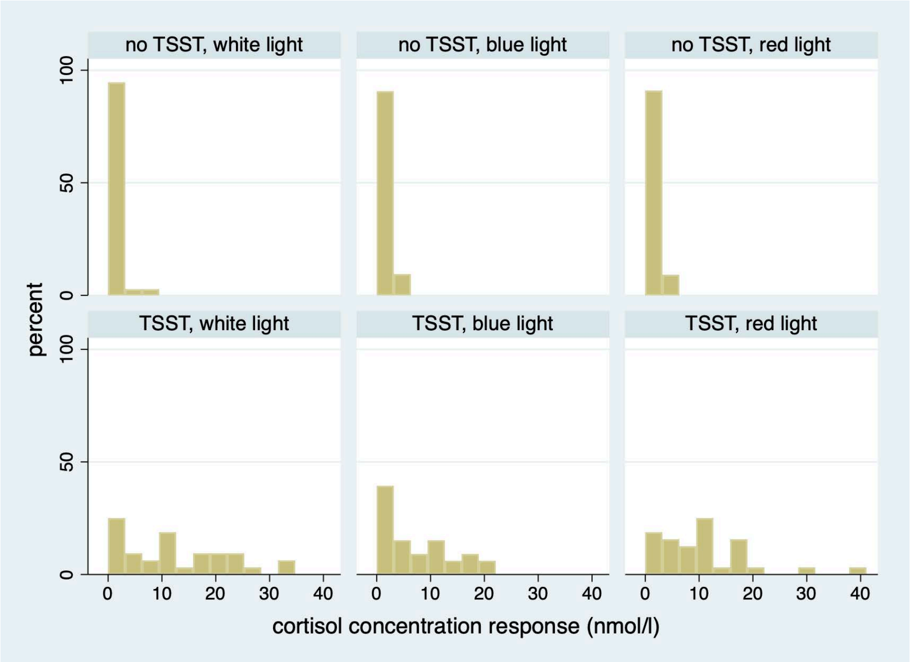

Fig 2. Cortisol concentration response with and without TSST.

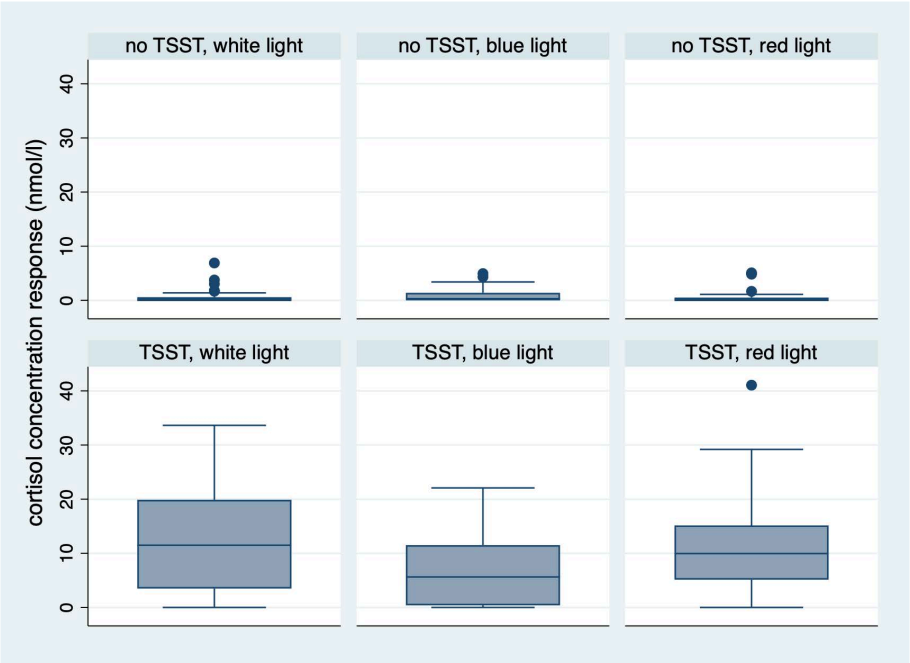

Fig 3. Cortisol concentration response with and without TSST by light color.

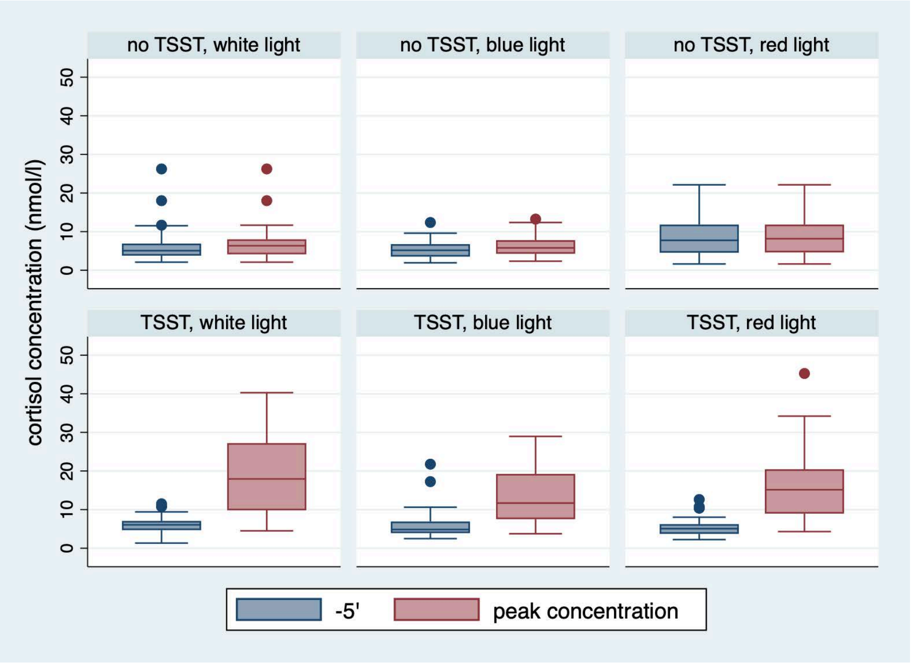

Supplement: S1 Appendix — (PDF) [file pone.0244881.s001.pdf]
