# Supplementary material for: Acute stress does not affect economic behavior in the experimental laboratory
Source: PLoS One. 2021 Jan 7;16(1):e0244881. doi: 10.1371/journal.pone.0244881 (PMC7790397; doi:10.1371/journal.pone.0244881)
Supplement: S2 Appendix — (PDF) [file pone.0244881.s002.pdf]

S2 Appendix - Stressor validation - TSST and self-reported wellness (stress, excitement, fatigue)

Fig 4. VAS reports on stress with and without TSST.

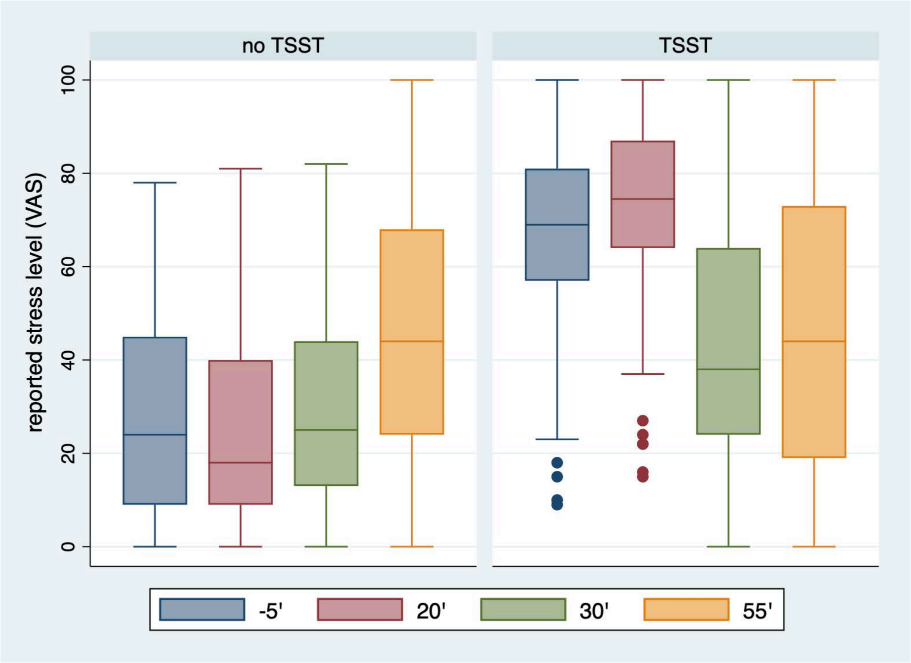

Fig 5. VAS reports on excitement with and without TSST.

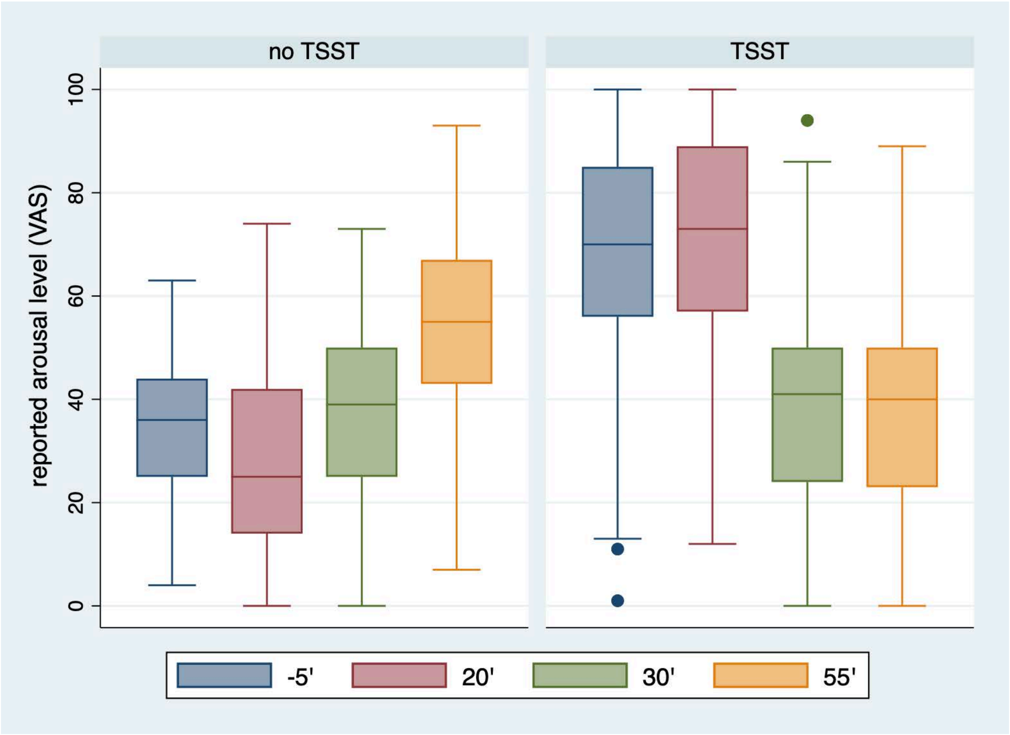

Fig 6. VAS reports on fatigue with and without TSST.

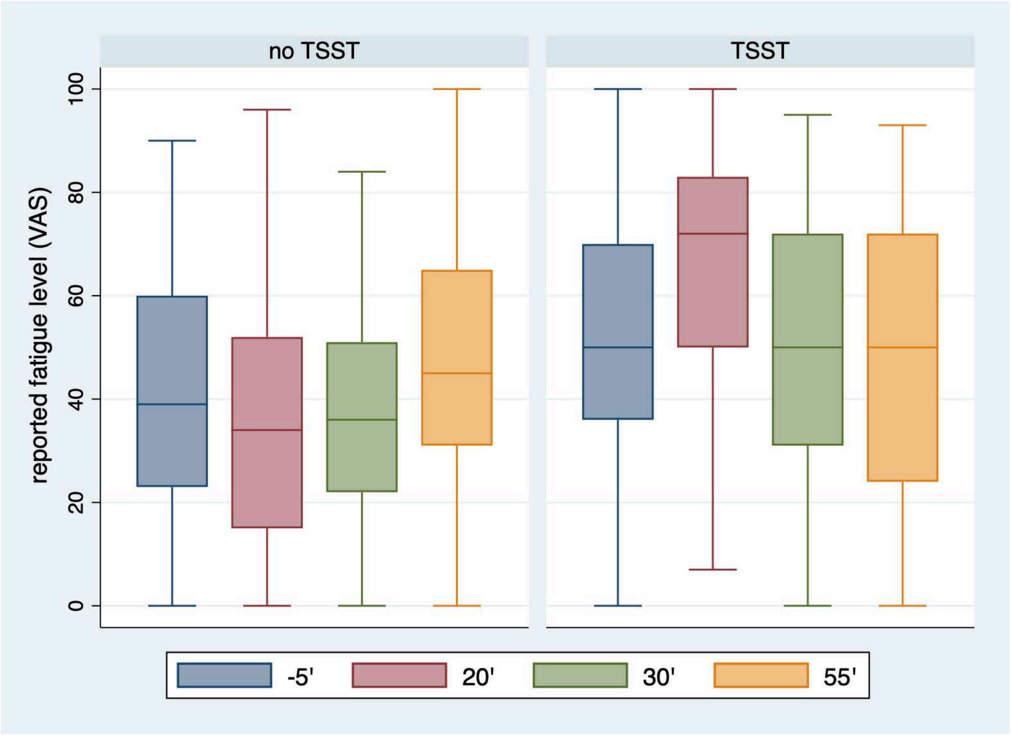

Table 1. Comparison for self-reported wellness (stress, excitement, fatigue) between no-TSST and TSST

	-5’	20’	30’	55’
stress				
p-value	0.000	0.000	0.001	0.943
benchmark mean	28.85	25.57	29.95	45.82
obs.	93	93	93	93
TSST mean	65.66	72.36	41.98	46.06
obs.	99	98	98	99
excitement				
p-value	0.000	0.000	0.596	0.000
benchmark mean	34.73	28.55	37.29	52.34
obs.	93	93	93	93
TSST mean	67.36	69.63	38.96	39.82
obs.	99	99	98	99
fatigue				
p-value	0.001	0.000	0.000	0.830
benchmark mean	39.42	35.59	37.62	48.03
obs.	93	93	93	93
TSST mean	51.19	65.70	50.71	48.52
obs.	99	99	98	99

Note: p-values for the Wilcoxon rank-sum test.
