# Supplementary material for: Acute stress does not affect economic behavior in the experimental laboratory
Source: PLoS One. 2021 Jan 7;16(1):e0244881. doi: 10.1371/journal.pone.0244881 (PMC7790397; doi:10.1371/journal.pone.0244881)
Supplement: S3 Appendix — (PDF) [file pone.0244881.s003.pdf]

S3 Appendix - Treatment effects by gender

Table 2. Stress and changes in behavior by gender (binary comparisons and treatment effects)

men	CRT	beauty contest	risk attitude declared	risk attitude lottery	risk attitude boxes	dictator game	gift exchange	joy of destruction
no TSST	1.177	37.44	4.871	1.484	38.89	735.6	0.242	272.8
obs.	62	62	62	62	62	62	62	62
TSST	1.544	33.72	4.228	1.351	43.93	792.1	0.123	343.0
obs.	57	57	57	57	57	57	57	57
p-value	0.0527	0.408	0.168	0.464	0.391	0.527	0.0945	0.596
Cohen's d	-0.351	0.210	0.241	0.247	-0.212	-0.202	0.308	-0.158
not stressed	1.222	37.06	4.937	1.444	40.08	735.2	0.254	284.4
obs.	63	63	63	63	63	63	63	63
stressed (2.5+)	1.547	33.40	4.245	1.453	43.38	800.9	0.113	331.1
obs.	53	53	53	53	53	53	53	53
p-value	0.0866	0.509	0.162	0.931	0.633	0.354	0.0541	0.792
Cohen's d	-0.312	0.206	0.263	-0.00851	-0.138	-0.235	0.362	-0.105
not stressed	1.241	36.50	5.017	1.414	40.50	746.0	0.259	269.2
obs.	58	58	58	58	58	58	58	58
stressed (1.5+)	1.500	34.28	4.224	1.483	42.67	784.5	0.121	342.2
obs.	58	58	58	58	58	58	58	58
p-value	0.164	0.827	0.104	0.678	0.900	0.635	0.0581	0.505
Cohen's d	-0.247	0.125	0.303	-0.0700	-0.0909	-0.137	0.354	-0.164

women	CRT	beauty contest	risk attitude declared	risk attitude lottery	risk attitude boxes	dictator game	gift exchange	joy of destruction
no TSST	0.903	37.48	4.484	1.452	37.71	750	0.194	322.6
obs.	31	31	31	31	31	31	31	31
TSST	0.857	34.86	4.310	1.048	39.52	792.6	0.119	250.6
obs.	42	42	42	42	42	42	42	42
p-value	0.909	0.573	0.703	0.104	0.775	0.338	0.379	0.705
Cohen's d	0.0471	0.140	0.0645	0.247	-0.0771	-0.183	0.206	0.175
not stressed	0.889	40.11	4.133	1.356	38.13	780.9	0.133	312.7
obs.	45	45	45	45	45	45	45	45
stressed (2.5+)	0.857	29.32	4.786	1	39.75	764.3	0.179	230.5
obs.	28	28	28	28	28	28	28	28
p-value	0.912	0.0328	0.294	0.197	0.837	0.579	0.599	0.550
Cohen's d	0.0325	0.600	-0.243	0.334	-0.0687	0.0709	-0.125	0.200
not stressed	0.900	39.65	4.200	1.375	37.12	787.2	0.125	308.8
obs.	40	40	40	40	40	40	40	40
stressed (1.5+)	0.848	31.52	4.606	1.030	40.73	759.1	0.182	247.7
obs.	33	33	33	33	33	33	33	33
p-value	0.771	0.126	0.506	0.207	0.653	0.449	0.499	0.857
Cohen's d	0.0527	0.444	-0.151	0.324	-0.153	0.120	-0.157	0.148

Note: p-values for a large-sample z-test of proportions (for gift exchange) and the Wilcoxon rank-sum test (for all other).
