# Supplementary material for: Acute stress does not affect economic behavior in the experimental laboratory
Source: PLoS One. 2021 Jan 7;16(1):e0244881. doi: 10.1371/journal.pone.0244881 (PMC7790397; doi:10.1371/journal.pone.0244881)
Supplement: S4 Appendix — (PDF) [file pone.0244881.s004.pdf]

S4 Appendix - Regression analysis

Note on measurement and scoring of some control variables.

- Answers to the 10 items in BFI were given on a 1-to-7 scale (1 - "not at all applicable", 7 - "very much applicable"). The scoring rules for our variables are as follows.
  - BFI extraversion: reverse score for question 6 + score for question 1.
  - BFI agreeableness: reverse score for question 2 + score for question 7.
  - BFI conscientiousness: reverse score for question 8 + score for question 3.
  - BFI neuroticism: reverse score for question 9 + score for question 4.
  - BFI openness to experience: reverse score for question 10 + score for question 5.
- Answers to the 40 items in PVQ were given on a 1-to-5 scale (1 - "not like me at all", 7 - "very much like me"). The scoring rules for our variables are as follows.
  - PVQ conformity: average score for questions 7, 16, 28, and 36.
  - PVQ tradition: average score for questions 9, 20, 25, and 38.
  - PVQ benevolence: average score for questions 12, 18, 27, and 33.
  - PVQ universalism: average score for questions 3, 8, 19, 23, 29, and 40.
  - PVQ self-direction: average score for questions 1, 11, 22, and 34.
  - PVQ stimulation: average score for questions 6, 15, and 30.
  - PVQ hedonism: average score for questions 10, 26, and 37.
  - PVQ achievement: average score for questions 4, 13, 24, and 32.
  - PVQ power: average score for questions 2, 17, and 39.
  - PVQ security: average score for questions 5, 14, 21, 31, and 35.
- Answers to the 20 items in CES-D were given on a 0-to-3 scale (0 - "rarely, never", 3 - "most of the time"). The scoring rule for our variable is as follows.
  - Scores for questions 4, 8, 12, and 16 were reversed.
  - Our variable is the aggregate score for all responses. Higher scores indicate greater depressive symptoms. A score of 16 or more is a sign of mild depression.
    [Refer to chapter 7 (Depression) in McDowell, I. (2006) *Measuring health: A guide to rating scales and questionnaires*, Oxford University Press.]
- Answers to the 20 items in STAI-T were given on a 1-to-4 scale (1 - "almost never", 4 - "almost always"). The scoring rule for our variable is as follows.
  - Scores for questions 1, 6, 7, 10, 13, 16, and 19 were reversed.
  - Our variable is the aggregate score for all responses. Higher scores indicate more anxiety. The cut-off score for relevant symptoms of anxiety is considered to be 39-40 by some, and 64-65 by others.
    [Refer to chapter 6 (Anxiety) in McDowell, I. (2006) *Measuring health: A guide to rating scales and questionnaires*, Oxford University Press, to Julian, L.J. (2011) "Measures of anxiety," *Arthritis Care Res (Hoboken)* 63: 1-11, and to Spielberger, C. D., Gorsuch, R. L., Lushene, R., Vagg, P. R., Jacobs, G. A. (1983) *Manual for the State-Trait Anxiety Inventory*, Palo Alto, CA: Consulting Psychologists Press.]

Table 3. Descriptive statistics for demographic and psychological control variables (part 1)

	Mean	Std. Dev.	25%	50%	75%	95%	Min.	Max.
age	20.9	2.12	19	21	22	24	18	35
female	0.38	0.49	0	0	1	1	0	1
college year	3.02	1.99	1	3	4	8	1	9
body height (cm)	167.5	8.45	160.5	169	173	180	148	190
body weight (kg)	58.7	10.2	51.6	58	65	75	40	98
BFI extraversion	8.07	3.08	6	8	11	13	2	14
BFI agreeableness	9.97	2.36	8	10	12	13	4	14
BFI conscientiousness	7.26	2.79	5	7	9	13	2	14
BFI neuroticism	7.99	2.56	6	8	10	12	2	13
BFI openness to experience	8.63	2.77	7	9	11	13	2	14
PVQ conformity	3.43	0.82	3	3.50	4	4.75	1	5
PVQ tradition	2.66	0.71	2.25	2.75	3.25	3.75	1	4.75
PVQ benevolence	3.44	0.75	3	3.50	4	4.75	1.50	5
PVQ universalism	3.76	0.63	3.33	3.83	4.17	4.67	1.83	5
PVQ self-direction	3.97	0.66	3.50	4	4.50	5	2	5
PVQ stimulation	3.60	0.92	3	3.67	4.33	5	1	5
PVQ hedonism	3.97	0.65	3.67	4	4.33	5	1.67	5
PVQ achievement	3.85	0.85	3.25	4	4.50	5	1.25	5
PVQ power	2.79	0.84	2.33	2.67	3.33	4.33	1	5
PVQ security	3.77	0.66	3.40	3.80	4.20	4.80	1.80	5
STAI-T	47.7	9.68	41	48	54	65	27	74
CES-D	16.4	9.96	9	14.5	22	35	0	53
Observations	192							

Table 4. Descriptive statistics for demographic and psychological control variables (part 2)

Waseda School	Freq.	Percent	Cum.
ASE - Advanced Science and Engineering	14	7.29	7.29
CMS - Culture, Media and Society	15	7.81	15.10
CSE - Creative Science and Engineering	6	3.12	18.23
EDU - Education	26	13.54	31.77
FSE - Fundamental Science and Engineering	6	3.12	34.90
HSS - Humanities and Social Sciences	18	9.38	44.27
HUM - Human Sciences	9	4.69	48.96
LAW - Law	17	8.85	57.81
PSE - Political Science and Economics	35	18.23	76.04
SILS - International Liberal Studies	9	4.69	80.73
SOC - Commerce	14	7.29	88.02
SPS - Sport Sciences	2	1.04	89.06
SSS - Social Sciences	13	6.77	95.83
other	8	4.17	100.00
Total	192	100.00	

Table 5. Descriptive statistics for experimental-procedure related control variables

	Freq.	Percent	Cum.
room 3-803	102	53.12	53.12
room 3-804	90	46.88	100.00
Total	192	100.00	
12/12/2018	28	14.58	14.58
09/01/2019	30	15.62	30.21
16/01/2019	17	8.85	39.06
19/01/2019	30	15.62	54.69
02/02/2019	27	14.06	68.75
25/02/2019	28	14.58	83.33
05/04/2019	32	16.67	100.00
Total	192	100.00	
section 1	62	32.29	32.29
section 2	66	34.38	66.67
section 3	64	33.33	100.00
Total	192	100.00	
blue light	66	34.38	34.38
no light	1	0.52	34.90
red light	56	29.17	64.06
white light	69	35.94	100.00
Total	192	100.00	

Table 6. Stress (TSST) and changes in behavior (regression analysis - complete table - part 1)

	CRT	beauty contest	risk attitude declared	risk attitude lottery	risk attitude boxes	dictator game	gift exchange	joy of destruction
TSST	-0.289	4.493	-0.482	-1.488***	8.280	-73.69	-1.676	1294.7
	(0.531)	(8.368)	(1.123)	(0.384)	(10.12)	(153.2)	(2.324)	(998.0)
female	-0.443	-7.288	-0.489	-0.141	0.241	-35.15	0.1000	1558.5**
	(0.280)	(5.319)	(0.824)	(0.243)	(6.458)	(111.8)	(0.970)	(720.7)
TSST × female	-0.197	-1.300	0.157	0.179	-9.774	73.31	-1.408	-1062.7
	(0.294)	(5.449)	(0.757)	(0.250)	(8.005)	(112.7)	(1.266)	(785.9)
college year	0.129**	0.0995	-0.239	-0.0280	0.596	81.00***	-0.190	-278.8
	(0.0604)	(1.102)	(0.181)	(0.0549)	(1.661)	(27.00)	(0.254)	(178.1)
Waseda SPS	0.0967	-8.288	-2.436	1.374**	42.37**	366.9*	0	-431.8
	(0.814)	(10.97)	(2.143)	(0.613)	(19.30)	(213.7)	(.)	(2048.0)
Waseda HUM	-0.129	-18.91	-0.638	0.883	12.21	346.2*	0.252	-2417.7
	(0.722)	(11.53)	(1.503)	(0.558)	(13.98)	(203.9)	(2.312)	(1494.6)
Waseda ASE	0.481	-35.64***	0.708	1.026**	-2.848	24.47	-0.675	-2936.2**
	(0.405)	(10.57)	(1.189)	(0.507)	(12.80)	(203.1)	(1.898)	(1255.6)
Waseda CSE	0.473	-28.99**	0.238	0.739	-7.688	366.4*	0.544	-819.0
	(0.449)	(12.27)	(1.477)	(0.508)	(10.91)	(210.4)	(2.112)	(1236.8)
Waseda SOC	0.522	-31.13***	-0.535	0.426	4.336	508.6**	0.208	-2232.4
	(0.423)	(11.39)	(1.244)	(0.569)	(14.38)	(197.2)	(2.015)	(1423.7)
Waseda SILS	0.0489	-29.33***	-1.304	0.509	-2.703	396.4	0.334	700.1
	(0.552)	(11.21)	(1.450)	(0.562)	(15.23)	(239.6)	(2.165)	(1398.7)
Waseda FSE	0.133	-31.73***	1.258	0.565	-2.023	49.11	3.387	-1023.9
	(0.591)	(11.02)	(1.789)	(0.658)	(15.84)	(224.3)	(2.414)	(1485.0)
Waseda PSE	0.108	-40.08***	0.863	0.844*	5.331	296.0	0.892	-429.7
	(0.388)	(10.01)	(1.102)	(0.511)	(11.16)	(184.2)	(1.721)	(1046.4)
Waseda EDU	0.343	-27.83***	-0.572	0.992**	-2.328	408.5**	0.874	-138.2
	(0.403)	(9.809)	(1.112)	(0.501)	(12.28)	(189.7)	(1.803)	(1108.3)
Waseda CMS	0.562	-31.89***	1.393	0.787	19.30	249.5	0.629	134.0
	(0.437)	(10.51)	(1.403)	(0.553)	(12.14)	(182.8)	(1.905)	(1207.4)
Waseda HSS	0.253	-24.22**	0.818	0.545	7.518	334.1*	1.145	-583.7
	(0.418)	(10.04)	(1.168)	(0.515)	(12.06)	(185.2)	(1.856)	(1060.1)
Waseda LAW	0.429	-25.47**	-0.0360	0.889*	6.916	440.5**	-0.831	-1183.6
	(0.411)	(10.03)	(1.318)	(0.519)	(12.32)	(200.2)	(1.982)	(1208.6)
Waseda SSS	0.575	-23.75**	-1.122	0.519	7.263	489.2**	-2.022	-1636.1
	(0.416)	(10.41)	(1.246)	(0.527)	(12.27)	(208.1)	(2.575)	(1279.7)
body height	-0.00663	-0.398	-0.0315	-0.00704	0.739*	-7.904	0.00795	80.07*
	(0.0151)	(0.281)	(0.0522)	(0.0129)	(0.433)	(6.246)	(0.0855)	(45.67)
body weight	0.00240	0.148	0.00510	-0.00246	-0.503*	4.672	-0.0254	-17.96
	(0.00919)	(0.185)	(0.0262)	(0.00761)	(0.283)	(4.219)	(0.0610)	(30.63)
...	...	...	...	...	...	...	...	...

Note: CRT, risk attitude (lottery): Poisson regression; beauty contest, risk attitude (boxes), dictator game, joy of destruction: TOBIT regression with robust standard errors; risk attitude (declared): OLS with robust standard errors; gift exchange: LOGIT regression with robust standard errors. Estimated coefficients significantly different from zero at *10%, **5%, and ***1%.

Table 7. Stress (TSST) and changes in behavior (regression analysis - complete table - part 2)

	CRT	beauty contest	risk attitude declared	risk attitude lottery	risk attitude boxes	dictator game	gift exchange	joy of destruction
...	...	...	...	...	...	...	...	...
room 3-804	-0.242	5.037	-0.291	-0.341**	-0.265	7.923	0.782	720.3*
	(0.178)	(3.331)	(0.547)	(0.165)	(4.442)	(74.61)	(0.721)	(429.4)
date 09/01/2019	0.345	0.374	1.414**	0.697**	-2.843	-67.01	-1.677	-898.8
	(0.259)	(4.612)	(0.694)	(0.298)	(7.153)	(107.6)	(1.070)	(632.0)
date 16/01/2019	0.517*	-5.392	1.691*	0.731**	-6.493	57.98	-1.598	-287.9
	(0.299)	(5.173)	(0.857)	(0.290)	(8.340)	(122.1)	(1.057)	(891.5)
date 19/01/2019	-0.0111	2.396	0.537	-1.222***	-14.71	-153.8	-2.836	920.7
	(0.554)	(8.811)	(1.329)	(0.369)	(11.36)	(175.0)	(2.056)	(1117.4)
date 02/02/2019	-0.599	12.82	0.690	-1.168***	-7.965	0.849	-5.212**	347.3
	(0.582)	(9.709)	(1.454)	(0.373)	(11.39)	(189.2)	(2.328)	(1160.1)
date 09/02/2019	0.333	4.868	0.125	-0.375	-17.11**	-0.0785	-3.182	-464.8
	(0.232)	(5.265)	(0.918)	(0.273)	(7.336)	(121.3)	(2.206)	(816.5)
date 05/04/2019	-0.385	3.725	0.552	-0.584*	3.994	-194.2	-2.737	412.4
	(0.430)	(6.468)	(0.989)	(0.315)	(8.351)	(135.0)	(2.190)	(785.8)
section 2	0.164	-1.619	0.443	0.193*	3.035	-49.05	-0.652	-1398.5***
	(0.152)	(3.182)	(0.497)	(0.111)	(4.341)	(63.04)	(0.771)	(476.1)
section 3	0.245	-3.431	0.235	-0.0396	5.611	30.09	0.867	-418.8
	(0.162)	(3.079)	(0.502)	(0.135)	(4.381)	(74.00)	(0.671)	(460.8)
blue light	-0.0681	7.994***	-0.584	-0.186	1.117	50.85	-0.188	779.1*
	(0.160)	(2.942)	(0.518)	(0.149)	(4.308)	(71.79)	(0.763)	(451.3)
red light	-0.560*	6.873	-0.626	-0.621***	-2.628	14.86	0.572	1159.4*
	(0.316)	(4.546)	(0.751)	(0.227)	(6.486)	(94.15)	(1.183)	(651.5)
BFI extroversion	-0.0164	0.194	0.0563	-0.0307	-0.353	-22.91**	0.0102	7.526
	(0.0278)	(0.485)	(0.0779)	(0.0224)	(0.693)	(11.05)	(0.107)	(68.20)
BFI agreeableness	-0.0752**	-0.361	-0.192*	-0.00114	-1.305	-17.57	0.125	98.06
	(0.0320)	(0.712)	(0.105)	(0.0271)	(0.875)	(15.95)	(0.163)	(100.7)
BFI conscientiousness	-0.0794***	0.798	0.00416	-0.0435*	1.636**	-2.539	0.191	-101.9
	(0.0306)	(0.569)	(0.0864)	(0.0249)	(0.751)	(12.82)	(0.131)	(81.69)
BFI neuroticism	0.0310	0.631	-0.206	-0.0184	-0.901	9.329	-0.0289	9.380
	(0.0415)	(0.751)	(0.127)	(0.0338)	(0.996)	(15.64)	(0.177)	(102.7)
BFI openness to experience	0.0231	-0.0247	0.0353	0.0307	0.853	15.31	-0.340**	-51.96
	(0.0327)	(0.634)	(0.115)	(0.0275)	(0.843)	(14.92)	(0.154)	(94.35)
...	...	...	...	...	...	...	...	...

Note: CRT, risk attitude (lottery): Poisson regression; beauty contest, risk attitude (boxes), dictator game, joy of destruction: TOBIT regression with robust standard errors; risk attitude (declared): OLS with robust standard errors; gift exchange: LOGIT regression with robust standard errors. Estimated coefficients significantly different from zero at *10%, **5%, and ***1%.

Table 8. Stress (TSST) and changes in behavior (regression analysis - complete table - part 3)

	CRT	beauty contest	risk attitude declared	risk attitude lottery	risk attitude boxes	dictator game	gift exchange	joy of destruction
...	...	...	...	...	...	...	...	...
PVQ conformity	0.214*	2.674	-0.322	-0.0526	2.616	33.99	-0.846*	0.319
	(0.110)	(2.490)	(0.328)	(0.0927)	(2.886)	(47.64)	(0.445)	(293.4)
PVQ tradition	-0.0944	1.559	0.330	0.100	-3.569	-103.9**	1.033*	31.24
	(0.108)	(2.111)	(0.326)	(0.0970)	(2.846)	(48.06)	(0.564)	(295.7)
PVQ benevolence	0.231**	-4.526**	-0.0596	0.0936	2.193	-78.71	0.204	-543.0
	(0.0962)	(1.980)	(0.291)	(0.0915)	(2.697)	(48.70)	(0.458)	(344.7)
PVQ universalism	-0.197	-2.496	0.784**	0.0512	1.948	-34.19	1.158*	-203.0
	(0.128)	(2.410)	(0.385)	(0.104)	(3.260)	(50.02)	(0.663)	(309.0)
PVQ self-direction	0.183	0.877	-0.130	-0.0638	-0.622	50.07	-0.106	-42.81
	(0.156)	(2.337)	(0.450)	(0.116)	(3.537)	(60.01)	(0.697)	(369.4)
PVQ stimulation	-0.0251	-0.651	0.737**	0.0156	0.541	-38.59	-0.732	316.8
	(0.101)	(1.695)	(0.304)	(0.0864)	(2.484)	(41.87)	(0.480)	(262.7)
PVQ hedonism	-0.142	-2.640	-0.160	-0.191*	2.319	63.50	1.592**	-558.4*
	(0.106)	(2.176)	(0.362)	(0.104)	(2.983)	(45.35)	(0.708)	(327.7)
PVQ achievement	-0.00544	2.951	0.529*	0.145*	4.322	8.650	1.597***	414.0
	(0.0973)	(2.066)	(0.302)	(0.0861)	(2.809)	(40.71)	(0.560)	(295.1)
PVQ power	0.126	-2.549	0.190	-0.0127	-3.095	37.91	-0.264	432.7
	(0.101)	(1.764)	(0.321)	(0.0887)	(2.693)	(42.11)	(0.405)	(283.4)
PVQ security	-0.200*	-1.368	-0.405	-0.126	-5.246	153.6***	-1.709***	323.2
	(0.115)	(2.311)	(0.429)	(0.101)	(3.318)	(53.98)	(0.604)	(325.1)
STAI-T	-0.00493	-0.210	-0.00301	-0.00644	-0.143	-3.400	-0.0743	-39.52
	(0.0138)	(0.286)	(0.0397)	(0.0123)	(0.327)	(5.411)	(0.0570)	(34.27)
CES-D	-0.0218**	-0.00743	0.0165	0.000304	0.0811	2.672	0.0634	18.38
	(0.00939)	(0.212)	(0.0351)	(0.00967)	(0.252)	(4.068)	(0.0483)	(28.20)
CRT		-1.766	-0.139	-0.0333	-1.501	68.07**	0.130	-79.85
		(1.159)	(0.215)	(0.0583)	(1.581)	(26.93)	(0.323)	(199.9)
Constant	4.872*	176.6***	6.135	2.608	-36.53	2184.1*	-14.42	-16756.7**
	(2.939)	(62.40)	(10.86)	(3.042)	(84.89)	(1278.2)	(14.29)	(8299.0)
Observations	191	191	191	191	191	191	189	191
R2			0.371					
Pseudo R2	0.101	0.0489		0.0779	0.0352	0.0387	0.358	0.0916
p-value: $\beta_{TSST} = 0$	0.587	0.592	0.669	0.000107	0.414	0.631	0.471	0.197
p-value: $\beta_{TSST} + \beta_{TSST \times female} = 0$	0.503	0.812	0.836	0.473	0.224	0.516	0.266	0.178

Note: CRT, risk attitude (lottery): Poisson regression; beauty contest, risk attitude (boxes), dictator game, joy of destruction: TOBIT regression with robust standard errors; risk attitude (declared): OLS with robust standard errors; gift exchange: LOGIT regression with robust standard errors. Estimated coefficients significantly different from zero at *10%, **5%, and ***1%.

Table 9. Stress (cortisol response 2.5+ nmol/l) and changes in behavior (regression analysis - shortened table)

	CRT	beauty contest	risk attitude declared	risk attitude lottery	risk attitude boxes	dictator game	gift exchange	joy of destruction
stressed (2.5+)	-0.0861	-6.181	0.442	-0.0684	3.767	-94.56	-0.321	478.0
	(0.224)	(3.899)	(0.634)	(0.205)	(5.777)	(100.3)	(0.920)	(677.0)
female	-0.487*	-6.134	-0.837	-0.0120	-0.961	2.656	-0.969	1333.9*
	(0.280)	(5.235)	(0.819)	(0.259)	(6.457)	(108.6)	(1.066)	(678.5)
stressed (2.5+) × female	-0.0564	-4.081	0.836	-0.287	-5.042	-38.85	1.219	-646.3
	(0.333)	(5.297)	(0.833)	(0.278)	(7.671)	(114.2)	(1.205)	(734.0)
controls	✓	✓	✓	✓	✓	✓	✓	✓
Observations	188	188	188	188	188	188	186	188
R2			0.382					
Pseudo R2	0.0997	0.0553		0.0599	0.0351	0.0416	0.343	0.0887
p-value: $\beta_{stressed} = 0$	0.700	0.115	0.487	0.739	0.515	0.347	0.727	0.481
p-value: $\beta_{stressed} + \beta_{stressed \times female} = 0$	0.866	0.442	0.317	0.302	0.512	0.734	0.312	0.380

Note: CRT, risk attitude (lottery): Poisson regression; beauty contest, risk attitude (boxes), dictator game, joy of destruction: TOBIT regression with robust standard errors; risk attitude (declared): OLS with robust standard errors; gift exchange: LOGIT regression with robust standard errors. Estimated coefficients significantly different from zero at *10%, **5%, and ***1%. Controls for demographics, session characteristics, cognitive skills and personality traits.

Fig 7. Predictive Margins of cortisol response (2.5+ nmol/l) by gender with 95% CIs.

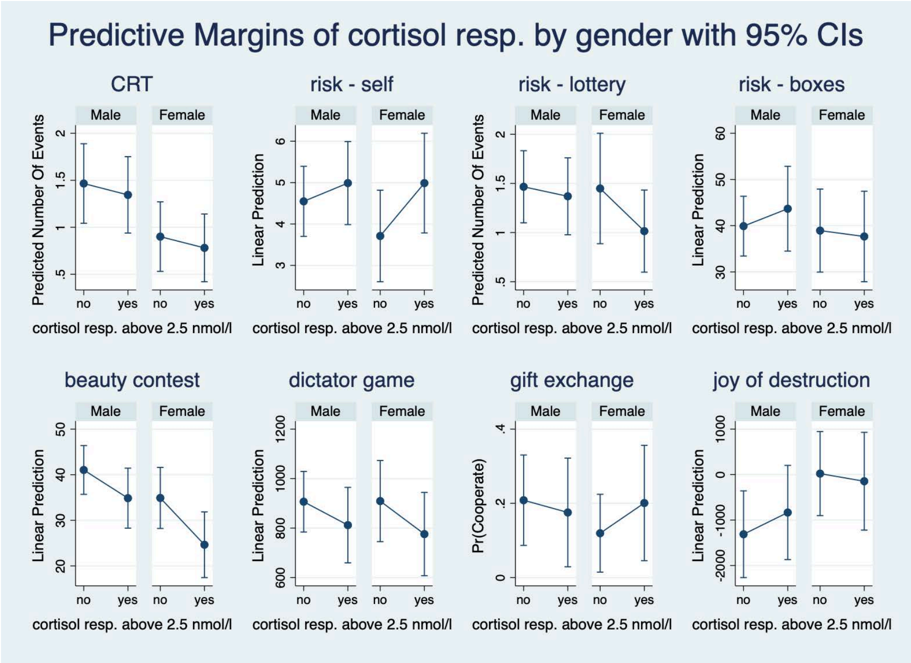

Table 10. Stress (cortisol response 2.5+ nmol/l) and changes in behavior (regression analysis - complete table - part 1)

	CRT	beauty		risk attitude		dictator	gift	joy of
		contest	declared	lottery	boxes	game	exchange	destruction
stressed (2.5+)	-0.0861	-6.181	0.442	-0.0684	3.767	-94.56	-0.321	478.0
	(0.224)	(3.899)	(0.634)	(0.205)	(5.777)	(100.3)	(0.920)	(677.0)
female	-0.487*	-6.134	-0.837	-0.0120	-0.961	2.656	-0.969	1333.9*
	(0.280)	(5.235)	(0.819)	(0.259)	(6.457)	(108.6)	(1.066)	(678.5)
stressed (2.5+) × female	-0.0564	-4.081	0.836	-0.287	-5.042	-38.85	1.219	-646.3
	(0.333)	(5.297)	(0.833)	(0.278)	(7.671)	(114.2)	(1.205)	(734.0)
college year	0.112*	0.158	-0.281	-0.0706	0.00218	88.17***	-0.223	-288.9
	(0.0620)	(1.104)	(0.183)	(0.0547)	(1.647)	(27.65)	(0.227)	(181.1)
Waseda SPS	0.216	-7.621	-2.311	1.471**	44.35**	344.0*	0	-269.5
	(0.826)	(10.26)	(1.966)	(0.660)	(19.01)	(206.5)	(.)	(2057.3)
Waseda HUM	-0.0473	-17.47	-0.939	0.929	13.79	348.3*	-0.120	-2273.1
	(0.722)	(10.76)	(1.510)	(0.595)	(13.52)	(203.8)	(2.238)	(1467.4)
Waseda ASE	0.604	-36.24***	0.808	1.248**	-1.341	-9.405	-0.323	-2775.6**
	(0.414)	(9.905)	(1.209)	(0.521)	(12.18)	(196.6)	(1.719)	(1177.8)
Waseda CSE	0.531	-30.00**	0.186	0.898*	-6.138	335.1	0.409	-718.9
	(0.453)	(12.18)	(1.427)	(0.531)	(10.61)	(216.0)	(1.977)	(1271.3)
Waseda SOC	0.600	-33.97***	-0.218	0.609	6.390	456.7**	0.288	-2137.2
	(0.433)	(11.22)	(1.255)	(0.581)	(13.98)	(200.2)	(1.859)	(1336.3)
Waseda SILS	0.117	-29.67***	-1.176	0.667	-2.138	393.9	0.321	800.6
	(0.543)	(11.05)	(1.458)	(0.572)	(14.80)	(239.1)	(1.982)	(1379.1)
Waseda FSE	0.277	-33.22***	1.393	0.893	0.950	18.52	3.368	-991.8
	(0.603)	(10.66)	(1.702)	(0.669)	(14.81)	(223.5)	(2.103)	(1401.2)
Waseda PSE	0.170	-41.69***	1.009	0.979*	6.730	290.8	1.016	-358.7
	(0.382)	(9.667)	(1.114)	(0.529)	(10.85)	(184.9)	(1.600)	(1023.1)
Waseda EDU	0.409	-28.56***	-0.571	1.051**	-0.972	381.2**	0.691	-137.6
	(0.408)	(9.523)	(1.125)	(0.523)	(11.87)	(190.7)	(1.634)	(1081.9)
Waseda CMS	0.614	-32.54***	1.346	0.929	20.11*	235.4	0.543	92.00
	(0.444)	(10.16)	(1.445)	(0.571)	(11.77)	(181.5)	(1.913)	(1187.2)
Waseda HSS	0.291	-26.02***	1.019	0.676	7.730	322.7*	1.254	-554.6
	(0.426)	(9.759)	(1.202)	(0.538)	(11.78)	(183.9)	(1.705)	(1048.7)
Waseda LAW	0.520	-25.00**	-0.0655	0.962*	8.489	405.0**	-0.685	-1154.6
	(0.420)	(9.593)	(1.338)	(0.540)	(11.85)	(202.7)	(1.876)	(1145.5)
Waseda SSS	0.704*	-25.87***	-0.913	0.724	11.04	450.9**	-1.505	-1444.3
	(0.417)	(9.884)	(1.208)	(0.543)	(11.69)	(208.3)	(2.418)	(1220.1)
body height	-0.00302	-0.407	-0.0385	-0.00278	0.839*	-7.673	0.00359	75.35*
	(0.0153)	(0.287)	(0.0512)	(0.0136)	(0.435)	(6.270)	(0.0886)	(45.00)
body weight	0.00307	0.141	0.00715	-0.00343	-0.517*	4.311	-0.00624	-18.54
	(0.00940)	(0.183)	(0.0259)	(0.00743)	(0.286)	(4.242)	(0.0566)	(30.47)
...	...	...	...	...	...	...	...	...

Note: CRT, risk attitude (lottery): Poisson regression; beauty contest, risk attitude (boxes), dictator game, joy of destruction: TOBIT regression with robust standard errors; risk attitude (declared): OLS with robust standard errors; gift exchange: LOGIT regression with robust standard errors. Estimated coefficients significantly different from zero at *10%, **5%, and ***1%.

Table 11. Stress (cortisol response 2.5+ nmol/l) and changes in behavior (regression analysis - complete table - part 2)

	CRT	beauty contest	risk attitude declared	lottery	boxes	dictator game	gift exchange	joy of destruction
...	...	...	...	...	...	...	...	...
room 3-804	-0.212	3.055	0.0741	0.0410	-2.237	13.42	1.238*	563.0
	(0.144)	(2.614)	(0.432)	(0.122)	(3.488)	(65.47)	(0.694)	(374.7)
date 09/01/2019	0.320	-0.00189	1.309*	0.341	-3.193	-43.93	-1.756	-954.2
	(0.222)	(4.314)	(0.686)	(0.254)	(7.192)	(110.7)	(1.126)	(627.4)
date 16/01/2019	0.354	-5.441	1.874**	0.303	-8.239	41.01	-1.757*	-234.0
	(0.248)	(4.894)	(0.874)	(0.279)	(8.242)	(125.2)	(1.034)	(857.5)
date 19/01/2019	0.289	-7.404*	1.486**	0.0735	-17.27***	-192.1*	-0.589	152.5
	(0.246)	(4.189)	(0.693)	(0.271)	(5.852)	(112.1)	(0.998)	(687.4)
date 02/02/2019	-0.295	3.362	1.669**	0.187	-10.84	-25.17	-2.364*	-339.2
	(0.298)	(5.680)	(0.823)	(0.275)	(6.976)	(119.1)	(1.412)	(700.2)
date 25/02/2019	0.400*	-0.684	0.688	-0.00261	-18.27***	-47.65	-1.933	-844.9
	(0.225)	(4.616)	(0.831)	(0.275)	(6.697)	(114.7)	(1.217)	(785.2)
date 05/04/2019	-0.268	-3.730	1.402	0.205	2.433	-237.6**	-1.157	-11.72
	(0.306)	(4.584)	(0.847)	(0.278)	(6.847)	(119.5)	(1.011)	(735.7)
section 2	0.134	-0.386	0.390	0.157	2.109	-63.69	-0.453	-1366.6***
	(0.155)	(3.116)	(0.497)	(0.124)	(4.299)	(63.89)	(0.783)	(476.8)
section 3	0.233	-2.840	0.230	0.0221	5.124	24.15	0.772	-461.1
	(0.164)	(2.985)	(0.496)	(0.142)	(4.346)	(72.84)	(0.688)	(451.5)
blue light	-0.108	7.276**	-0.322	-0.0658	0.113	55.26	-0.239	795.4*
	(0.156)	(2.869)	(0.498)	(0.145)	(4.347)	(69.80)	(0.818)	(453.8)
red light	-0.411*	4.512	-0.373	-0.0175	-2.397	19.85	1.172	1002.7*
	(0.211)	(3.437)	(0.607)	(0.168)	(4.970)	(79.89)	(1.118)	(554.4)
BFI extroversion	-0.0196	0.302	0.0462	-0.0367*	-0.518	-23.22**	0.0354	7.639
	(0.0283)	(0.448)	(0.0763)	(0.0223)	(0.688)	(11.27)	(0.112)	(70.51)
BFI agreeableness	-0.0733**	-0.605	-0.184*	-0.00123	-1.164	-21.18	0.124	89.13
	(0.0328)	(0.701)	(0.102)	(0.0296)	(0.871)	(16.60)	(0.156)	(98.96)
BFI conscientiousness	-0.0855***	0.596	0.0630	-0.0341	1.478*	-2.666	0.256**	-90.32
	(0.0331)	(0.597)	(0.0877)	(0.0250)	(0.811)	(12.90)	(0.128)	(82.25)
BFI neuroticism	0.0271	0.408	-0.175	-0.0286	-0.777	9.001	0.00364	29.93
	(0.0409)	(0.717)	(0.126)	(0.0343)	(0.971)	(15.37)	(0.187)	(102.5)
BFI openness to experience	0.0200	0.257	-0.00337	0.00435	0.745	15.62	-0.374**	-52.75
	(0.0342)	(0.599)	(0.108)	(0.0289)	(0.813)	(14.91)	(0.149)	(91.54)
...	...	...	...	...	...	...	...	...

Note: CRT, risk attitude (lottery): Poisson regression; beauty contest, risk attitude (boxes), dictator game, joy of destruction: TOBIT regression with robust standard errors; risk attitude (declared): OLS with robust standard errors; gift exchange: LOGIT regression with robust standard errors. Estimated coefficients significantly different from zero at *10%, **5%, and ***1%.

Table 12. Stress (cortisol response 2.5+ nmol/l) and changes in behavior (regression analysis - complete table - part 3)

	CRT	beauty contest	risk attitude declared	risk attitude lottery	risk attitude boxes	dictator game	gift exchange	joy of destruction
...	...	...	...	...	...	...	...	...
PVQ conformity	0.182*	4.250*	-0.500*	-0.131	1.664	60.27	-0.927**	-2.526
	(0.109)	(2.253)	(0.283)	(0.0934)	(2.754)	(48.02)	(0.460)	(291.4)
PVQ tradition	-0.0736	1.415	0.285	0.0863	-3.307	-121.2**	0.940	26.90
	(0.110)	(2.053)	(0.320)	(0.0973)	(2.844)	(46.83)	(0.624)	(288.8)
PVQ benevolence	0.214**	-4.535**	-0.114	0.0453	1.445	-71.59	0.151	-587.4*
	(0.0979)	(1.969)	(0.293)	(0.0977)	(2.666)	(48.24)	(0.493)	(351.4)
PVQ universalism	-0.190	-2.377	0.900**	0.127	2.938	-51.61	1.117*	-108.0
	(0.131)	(2.388)	(0.387)	(0.110)	(3.331)	(51.44)	(0.572)	(318.1)
PVQ self-direction	0.143	2.394	-0.195	-0.0975	-1.336	72.48	-0.0816	-25.38
	(0.148)	(2.268)	(0.446)	(0.120)	(3.619)	(60.69)	(0.670)	(370.9)
PVQ stimulation	0.0000629	-2.357	0.814***	0.0389	1.816	-50.44	-0.699*	342.3
	(0.105)	(1.753)	(0.302)	(0.0904)	(2.626)	(43.90)	(0.412)	(272.8)
PVQ hedonism	-0.149	-2.664	-0.120	-0.136	2.434	75.60*	1.414**	-563.5*
	(0.111)	(2.248)	(0.367)	(0.105)	(3.048)	(45.51)	(0.650)	(333.0)
PVQ achievement	0.0109	3.015	0.387	0.124	3.174	1.428	1.475***	248.6
	(0.0985)	(2.057)	(0.296)	(0.0877)	(2.849)	(41.14)	(0.491)	(308.5)
PVQ power	0.127	-2.580	0.237	-0.00935	-2.159	26.54	-0.274	473.7*
	(0.101)	(1.755)	(0.320)	(0.0887)	(2.654)	(41.41)	(0.338)	(281.2)
PVQ security	-0.164	-1.945	-0.355	-0.0416	-4.412	155.4***	-1.561**	344.8
	(0.115)	(2.217)	(0.415)	(0.103)	(3.242)	(54.51)	(0.613)	(326.6)
STAI-T	-0.00188	-0.258	-0.00598	-0.00305	-0.0491	-3.541	-0.0627	-37.35
	(0.0138)	(0.278)	(0.0391)	(0.0119)	(0.324)	(5.397)	(0.0578)	(33.03)
CES-D	-0.0255***	0.0675	0.0242	0.000674	-0.0543	3.707	0.0676	20.71
	(0.00943)	(0.202)	(0.0347)	(0.00984)	(0.260)	(4.136)	(0.0446)	(28.40)
CRT		-1.502	-0.0937	-0.0142	-1.585	68.24**	0.175	-33.34
		(1.144)	(0.214)	(0.0608)	(1.613)	(27.47)	(0.292)	(197.6)
Constant	3.677	197.4***	4.781	-0.312	-64.82	2479.8*	-16.82	-15688.2*
	(3.025)	(61.34)	(10.09)	(2.913)	(81.26)	(1289.5)	(15.63)	(8287.8)
Observations	188	188	188	188	188	188	186	188
R2			0.382					
Pseudo R2	0.0997	0.0553		0.0599	0.0351	0.0416	0.343	0.0887
p-value: $\beta_{stressed} = 0$	0.700	0.115	0.487	0.739	0.515	0.347	0.727	0.481
p-value: $\beta_{stressed} + \beta_{stressed \times female} = 0$	0.866	0.442	0.317	0.302	0.512	0.734	0.312	0.380

Note: CRT, risk attitude (lottery): Poisson regression; beauty contest, risk attitude (boxes), dictator game, joy of destruction: TOBIT regression with robust standard errors; risk attitude (declared): OLS with robust standard errors; gift exchange: LOGIT regression with robust standard errors. Estimated coefficients significantly different from zero at *10%, **5%, and ***1%.

Table 13. Stress (cortisol response 1.5+ nmol/l) and changes in behavior (regression analysis - shortened table)

	CRT	beauty contest	risk attitude declared	risk attitude lottery	risk attitude boxes	dictator game	gift exchange	joy of destruction
stressed (1.5+)	-0.174	-3.331	0.156	0.0607	7.823	-192.5*	-0.533	1061.2
	(0.221)	(4.033)	(0.622)	(0.188)	(5.552)	(97.47)	(0.819)	(668.4)
female	-0.538*	-6.865	-0.677	0.0693	0.423	-20.23	-1.156	1446.2**
	(0.285)	(5.519)	(0.832)	(0.256)	(6.568)	(107.1)	(1.112)	(697.2)
stressed (1.5+) × female	0.0810	-2.515	0.447	-0.474	-8.585	40.86	1.685	-957.7
	(0.322)	(5.388)	(0.894)	(0.293)	(8.215)	(115.9)	(1.355)	(740.0)
controls	✓	✓	✓	✓	✓	✓	✓	✓
Observations	188	188	188	188	188	188	186	188
R2			0.371					
Pseudo R2	0.100	0.0527		0.0619	0.0359	0.0434	0.347	0.0916
p-value: $\beta_{stressed} = 0$	0.431	0.410	0.802	0.747	0.161	0.0502	0.515	0.115
p-value: $\beta_{stressed} + \beta_{stressed \times female} = 0$	0.801	0.641	0.618	0.106	0.298	0.725	0.214	0.198

Note: CRT, risk attitude (lottery): Poisson regression; beauty contest, risk attitude (boxes), dictator game, joy of destruction: TOBIT regression with robust standard errors; risk attitude (declared): OLS with robust standard errors; gift exchange: LOGIT regression with robust standard errors. Estimated coefficients significantly different from zero at *10%, **5%, and ***1%. Controls for demographics, session characteristics, cognitive skills and personality traits.

Fig 8. Predictive Margins of cortisol response (1.5+ nmol/l) by gender with 95% CIs.

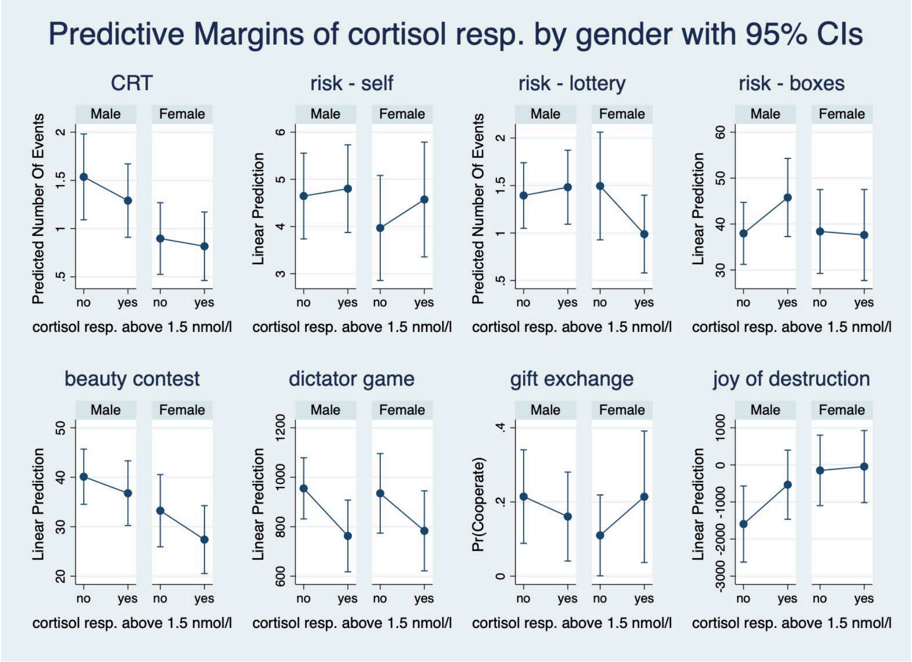

Table 14. Stress (cortisol response 1.5+ nmol/l) and changes in behavior (regression analysis - complete table - part 1)

	CRT	beauty contest	risk attitude declared	risk attitude lottery	risk attitude boxes	dictator game	gift exchange	joy of destruction
stressed (1.5+)	-0.174	-3.331	0.156	0.0607	7.823	-192.5*	-0.533	1061.2
	(0.221)	(4.033)	(0.622)	(0.188)	(5.552)	(97.47)	(0.819)	(668.4)
female	-0.538*	-6.865	-0.677	0.0693	0.423	-20.23	-1.156	1446.2**
	(0.285)	(5.519)	(0.832)	(0.256)	(6.568)	(107.1)	(1.112)	(697.2)
stressed (1.5+) × female	0.0810	-2.515	0.447	-0.474	-8.585	40.86	1.685	-957.7
	(0.322)	(5.388)	(0.894)	(0.293)	(8.215)	(115.9)	(1.355)	(740.0)
college year	0.112*	0.228	-0.289	-0.0701	0.0588	87.73***	-0.219	-293.2
	(0.0621)	(1.099)	(0.179)	(0.0541)	(1.662)	(27.14)	(0.227)	(178.6)
Waseda SPS	0.293	-5.000	-2.619	1.582***	43.30**	452.2**	0	-569.7
	(0.804)	(10.62)	(2.186)	(0.612)	(19.19)	(224.4)	(.)	(2046.5)
Waseda HUM	-0.0415	-18.50*	-0.806	0.896	13.29	353.5*	0.0108	-2406.8
	(0.725)	(11.06)	(1.496)	(0.588)	(13.60)	(197.4)	(2.209)	(1456.8)
Waseda ASE	0.604	-36.45***	0.814	1.217**	-1.584	-7.669	-0.291	-2884.5**
	(0.412)	(10.06)	(1.172)	(0.517)	(12.33)	(195.1)	(1.735)	(1174.4)
Waseda CSE	0.567	-28.65**	0.138	0.919*	-7.792	389.5*	0.540	-1161.9
	(0.440)	(12.07)	(1.436)	(0.525)	(10.51)	(205.1)	(2.037)	(1235.2)
Waseda SOC	0.596	-33.59***	-0.290	0.571	6.568	441.9**	0.422	-2129.0
	(0.430)	(11.34)	(1.230)	(0.580)	(14.08)	(196.3)	(1.895)	(1340.7)
Waseda SILS	0.134	-29.73***	-1.182	0.644	-2.348	396.6*	0.415	718.8
	(0.541)	(11.16)	(1.432)	(0.563)	(14.85)	(237.2)	(2.019)	(1362.4)
Waseda FSE	0.307	-32.86***	1.385	0.860	-0.590	53.90	3.486*	-1261.1
	(0.596)	(10.90)	(1.712)	(0.662)	(15.34)	(217.1)	(2.115)	(1433.3)
Waseda PSE	0.167	-41.23***	0.952	0.998*	6.952	296.8	1.038	-402.4
	(0.386)	(9.802)	(1.075)	(0.521)	(10.91)	(182.3)	(1.608)	(1017.7)
Waseda EDU	0.411	-28.59***	-0.562	1.026**	-1.328	385.9**	0.757	-228.4
	(0.406)	(9.614)	(1.096)	(0.520)	(12.00)	(189.1)	(1.633)	(1071.0)
Waseda CMS	0.606	-32.82***	1.390	0.918	20.25*	226.2	0.590	46.48
	(0.443)	(10.34)	(1.410)	(0.563)	(11.88)	(177.3)	(1.968)	(1168.8)
Waseda HSS	0.306	-24.72**	0.887	0.720	7.678	340.4*	1.202	-597.6
	(0.420)	(9.832)	(1.161)	(0.536)	(11.82)	(179.9)	(1.722)	(1032.7)
Waseda LAW	0.543	-25.39**	-0.0381	0.906*	7.571	422.1**	-0.577	-1311.9
	(0.420)	(9.723)	(1.306)	(0.536)	(12.05)	(199.5)	(1.891)	(1147.4)
Waseda SSS	0.709*	-26.17***	-0.878	0.704	10.89	450.3**	-1.395	-1542.4
	(0.423)	(9.991)	(1.185)	(0.539)	(11.82)	(204.2)	(2.402)	(1209.5)
body height	-0.00421	-0.479*	-0.0311	-0.00416	0.866**	-8.817	0.0122	78.18*
	(0.0155)	(0.285)	(0.0520)	(0.0134)	(0.428)	(6.125)	(0.0953)	(43.71)
body weight	0.00498	0.173	0.00408	-0.00369	-0.578**	5.875	-0.00593	-26.92
	(0.00963)	(0.185)	(0.0272)	(0.00777)	(0.276)	(4.378)	(0.0581)	(28.95)
...	...	...	...	...	...	...	...	...

Note: CRT, risk attitude (lottery): Poisson regression; beauty contest, risk attitude (boxes), dictator game, joy of destruction: TOBIT regression with robust standard errors; risk attitude (declared): OLS with robust standard errors; gift exchange: LOGIT regression with robust standard errors. Estimated coefficients significantly different from zero at *10%, **5%, and ***1%. Controls for demographics, session characteristics, cognitive skills and personality traits.

Table 15. Stress (cortisol response 1.5+ nmol/l) and changes in behavior (regression analysis - complete table - part 2)

	CRT	beauty contest	risk attitude declared	risk attitude lottery	risk attitude boxes	dictator game	gift exchange	joy of destruction
...	...	...	...	...	...	...	...	...
room 3-804	-0.222	3.902	-0.0315	0.0552	-1.534	-1.823	1.221*	687.8*
	(0.136)	(2.603)	(0.431)	(0.123)	(3.467)	(63.81)	(0.679)	(378.0)
date 09/01/2019	0.338	0.0115	1.269*	0.299	-3.799	-34.76	-1.660	-1039.7*
	(0.220)	(4.304)	(0.693)	(0.258)	(7.113)	(109.4)	(1.138)	(620.4)
date 16/01/2019	0.383	-4.700	1.749*	0.242	-8.908	43.12	-1.571	-261.1
	(0.248)	(4.835)	(0.900)	(0.283)	(8.479)	(126.6)	(1.090)	(870.2)
date 19/01/2019	0.262	-4.692	1.162*	0.138	-15.52***	-232.8**	-0.721	450.7
	(0.233)	(4.068)	(0.677)	(0.255)	(5.777)	(104.5)	(0.949)	(650.3)
date 02/02/2019	-0.335	5.631	1.390*	0.225	-8.920	-79.13	-2.427*	-24.56
	(0.296)	(5.709)	(0.820)	(0.269)	(7.164)	(113.9)	(1.458)	(703.3)
date 25/02/2019	0.396*	0.644	0.493	-0.0328	-17.75**	-75.88	-1.730	-714.3
	(0.216)	(4.819)	(0.868)	(0.270)	(6.843)	(114.5)	(1.294)	(808.3)
date 05/04/2019	-0.288	-1.421	1.119	0.242	3.687	-269.6**	-1.138	202.9
	(0.312)	(4.710)	(0.860)	(0.264)	(6.671)	(117.1)	(1.020)	(714.2)
section 2	0.137	-0.109	0.358	0.160	1.935	-51.51	-0.527	-1410.1***
	(0.155)	(3.207)	(0.502)	(0.123)	(4.253)	(64.66)	(0.807)	(472.8)
section 3	0.220	-3.048	0.252	0.0309	5.417	21.60	0.714	-448.7
	(0.163)	(3.002)	(0.505)	(0.144)	(4.354)	(72.99)	(0.686)	(451.7)
blue light	-0.103	7.828***	-0.384	-0.0590	0.0545	58.20	-0.219	791.2*
	(0.156)	(2.907)	(0.505)	(0.144)	(4.290)	(70.54)	(0.802)	(444.6)
red light	-0.427**	4.924	-0.393	0.0351	-1.753	13.77	0.995	1087.5*
	(0.211)	(3.535)	(0.617)	(0.164)	(4.886)	(79.58)	(1.105)	(557.3)
BFI extroversion	-0.0209	0.280	0.0467	-0.0385*	-0.482	-24.63**	0.0362	14.18
	(0.0285)	(0.453)	(0.0771)	(0.0226)	(0.687)	(11.09)	(0.114)	(70.08)
BFI agreeableness	-0.0756**	-0.546	-0.189*	0.00263	-1.078	-23.97	0.119	99.20
	(0.0326)	(0.711)	(0.102)	(0.0292)	(0.872)	(16.53)	(0.155)	(97.46)
BFI conscientiousness	-0.0854***	0.733	0.0427	-0.0363	1.504*	-3.767	0.264**	-80.42
	(0.0321)	(0.593)	(0.0874)	(0.0251)	(0.808)	(12.86)	(0.129)	(79.57)
BFI neuroticism	0.0267	0.472	-0.186	-0.0282	-0.736	8.138	0.0238	36.10
	(0.0419)	(0.727)	(0.127)	(0.0337)	(0.970)	(15.40)	(0.201)	(101.4)
BFI openness to experience	0.0231	0.234	0.000572	0.00548	0.668	18.54	-0.384**	-68.34
	(0.0342)	(0.605)	(0.110)	(0.0283)	(0.814)	(14.92)	(0.153)	(92.35)
...	...	...	...	...	...	...	...	...

Note: CRT, risk attitude (lottery): Poisson regression; beauty contest, risk attitude (boxes), dictator game, joy of destruction: TOBIT regression with robust standard errors; risk attitude (declared): OLS with robust standard errors; gift exchange: LOGIT regression with robust standard errors. Estimated coefficients significantly different from zero at *10%, **5%, and ***1%. Controls for demographics, session characteristics, cognitive skills and personality traits.

Table 16. Stress (cortisol response 1.5+ nmol/l) and changes in behavior (regression analysis - complete table - part 3)

	CRT	beauty contest	risk attitude declared	risk attitude lottery	risk attitude boxes	dictator game	gift exchange	joy of destruction
...	...	...	...	...	...	...	...	...
PVQ conformity	0.180*	3.762	-0.444	-0.124	1.792	60.08	-0.949**	-24.19
	(0.105)	(2.343)	(0.295)	(0.0912)	(2.774)	(47.45)	(0.463)	(281.9)
PVQ tradition	-0.0758	1.416	0.285	0.0786	-3.300	-122.8**	0.945	46.28
	(0.110)	(2.098)	(0.321)	(0.0997)	(2.820)	(47.13)	(0.624)	(289.9)
PVQ benevolence	0.223**	-4.372**	-0.137	0.0338	1.133	-62.58	0.198	-595.4*
	(0.0981)	(1.967)	(0.299)	(0.0981)	(2.619)	(48.07)	(0.527)	(347.5)
PVQ universalism	-0.195	-2.549	0.922**	0.125	3.108	-61.61	1.089*	-75.46
	(0.130)	(2.428)	(0.388)	(0.110)	(3.315)	(51.32)	(0.570)	(317.5)
PVQ self-direction	0.141	1.748	-0.126	-0.116	-1.557	73.75	-0.0546	-59.31
	(0.151)	(2.288)	(0.451)	(0.117)	(3.575)	(59.19)	(0.676)	(362.9)
PVQ stimulation	-0.00602	-2.027	0.781**	0.0578	2.193	-57.14	-0.729*	391.3
	(0.104)	(1.724)	(0.307)	(0.0876)	(2.630)	(43.62)	(0.409)	(270.3)
PVQ hedonism	-0.168	-2.845	-0.0973	-0.122	2.960	65.46	1.382**	-495.8
	(0.116)	(2.273)	(0.382)	(0.105)	(3.110)	(45.82)	(0.638)	(337.5)
PVQ achievement	0.0117	3.202	0.365	0.120	3.139	3.921	1.464***	239.7
	(0.0986)	(2.121)	(0.297)	(0.0879)	(2.819)	(40.25)	(0.487)	(304.4)
PVQ power	0.126	-2.731	0.253	-0.0196	-2.200	23.95	-0.252	478.4*
	(0.0996)	(1.762)	(0.321)	(0.0912)	(2.661)	(41.18)	(0.338)	(280.7)
PVQ security	-0.158	-1.899	-0.349	-0.0240	-4.511	160.1***	-1.590**	313.4
	(0.114)	(2.265)	(0.422)	(0.102)	(3.237)	(54.14)	(0.625)	(323.8)
STAI-T	-0.00122	-0.282	-0.00326	-0.00552	-0.0849	-3.366	-0.0598	-40.02
	(0.0139)	(0.283)	(0.0395)	(0.0118)	(0.326)	(5.443)	(0.0599)	(32.79)
CES-D	-0.0256***	0.103	0.0202	0.00263	-0.0400	4.000	0.0621	20.93
	(0.00965)	(0.206)	(0.0353)	(0.00985)	(0.259)	(4.140)	(0.0449)	(27.91)
CRT		-1.463	-0.101	-0.0105	-1.501	66.79**	0.154	-24.43
		(1.159)	(0.218)	(0.0602)	(1.606)	(27.10)	(0.289)	(197.4)
Constant	3.897	205.9***	3.999	-0.0445	-70.72	2769.7**	-18.61	-16622.1**
	(3.032)	(61.92)	(10.36)	(2.874)	(80.92)	(1272.8)	(17.01)	(8259.0)
Observations	188	188	188	188	188	188	186	188
R2			0.371					
Pseudo R2	0.100	0.0527		0.0619	0.0359	0.0434	0.347	0.0916
p-value: $\beta_{stressed} = 0$	0.431	0.410	0.802	0.747	0.161	0.0502	0.515	0.115
p-value: $\beta_{stressed} + \beta_{stressed \times female} = 0$	0.801	0.641	0.618	0.106	0.298	0.725	0.214	0.198

Note: CRT, risk attitude (lottery): Poisson regression; beauty contest, risk attitude (boxes), dictator game, joy of destruction: TOBIT regression with robust standard errors; risk attitude (declared): OLS with robust standard errors; gift exchange: LOGIT regression with robust standard errors. Estimated coefficients significantly different from zero at *10%, **5%, and ***1%. Controls for demographics, session characteristics, cognitive skills and personality traits.
