# Supplementary material for: Acute stress does not affect economic behavior in the experimental laboratory
Source: PLoS One. 2021 Jan 7;16(1):e0244881. doi: 10.1371/journal.pone.0244881 (PMC7790397; doi:10.1371/journal.pone.0244881)
Supplement: S5 Appendix — (PDF) [file pone.0244881.s005.pdf]

S5 Appendix - Additional procedural details and instructions

General instructions at the start of the experiment

You will be asked to take a saliva sample later on. Please keep your saliva in your mouth as you listen to my instructions. There is no need to do anything to produce more saliva; just keep it in your month.

The purpose of today's experiment is to understand the relationship between decision-making and some biological responses. We are going to analyze your stress level using endocrine substances in your saliva.

The experiment will last for about 90 minutes, and you will be divided into two groups. Those with ID number 1 to 5 will move to a different room to complete a task as a group. Those with ID number 6 to 10 will stay in this room and engage in a different task.

After you are done with the task, you will move to our experiment laboratory where you will be asked to accomplish various tasks on a computer screen. Finally, you will fill out more questionnaires, and when you are done, you will get paid and the experiment will end. During this experiment, you will also be asked to take five saliva samples, one of which is a practice. We will ask you a set of short questions each time you take saliva samples.

In order take a saliva sample, please pour your saliva into this tube using a straw provided. The equipment is all new and sanitized. Now we will practice taking a saliva sample. Pour you saliva into the tube. Please do not blow your saliva into it.

Your participation in this experiment is based on your free will, and you may withdraw from the experiment at any point. All of your personal information will be used only for the purpose of the experiment and will be kept strictly confidential. If you agree to participate in the experiment based on these conditions, please sign the consent form on the table in front of you. If you change your mind later on, please submit the withdrawal of the consent form that is also on the table to the Principal Investigator, and we will destroy all of your data. Please take the withdrawal of the consent form home with you.

zTree screens

The following figures display the (the original Japanese) zTree screens that participants were shown. All our zTree files are available upon request. Screens reporting the outcome of interactions and monetary gains are omitted. The note below each screenshot contains the English translation.

Fig 9. zTree screen for general instructions.

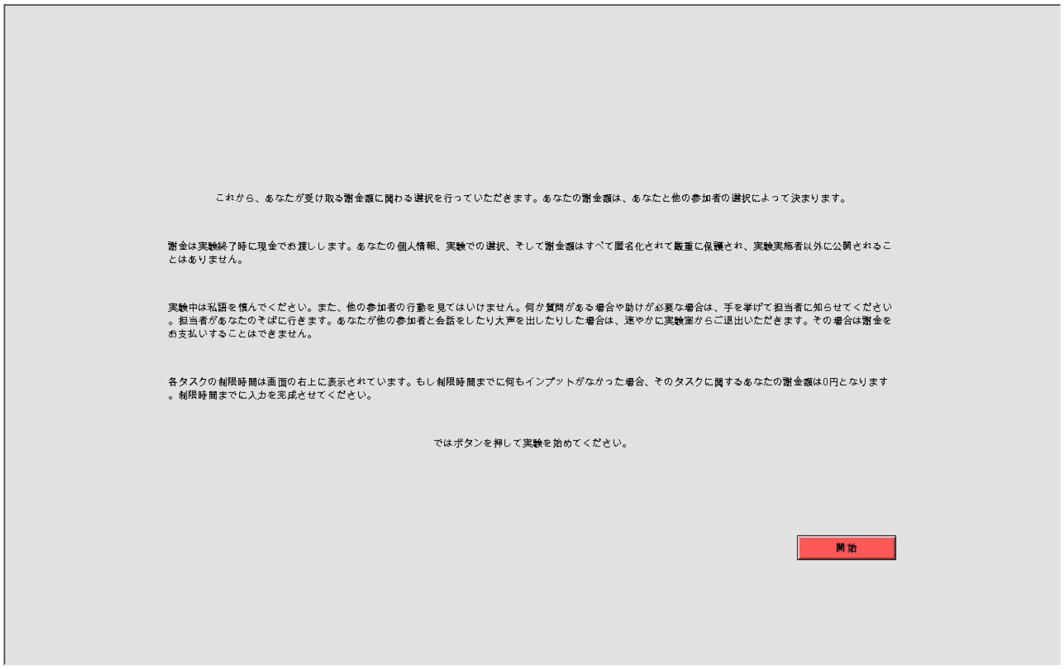

Translation:

In what follows, you will be asked to make a series of decisions that will allow you to earn money. Your earnings will depend on the decisions you make and the decisions of others.

Your earnings will be payed to you in cash at the end of the experiment. All your personal information, decisions and earnings in the experiment will be kept strictly anonymous and confidential, and will not be disclosed to anyone other than the experimenter.

Please refrain from talking during the experiment and looking at other participants' work. If you have any questions or need assistance of any kind, please raise your hand, and an experimenter will come to you. If you talk, exclaim out loud, etc., you will be asked to leave and you will forfeit your earnings.

The time limit for each task is displayed in the upper right corner of the screen. If you do not make an input by the time limit, your earnings for the task will be 0 yen. Please complete each task by the time limit.

Now click on the button to start the experiment.

Fig 10. zTree screen for CRT instructions and questions.

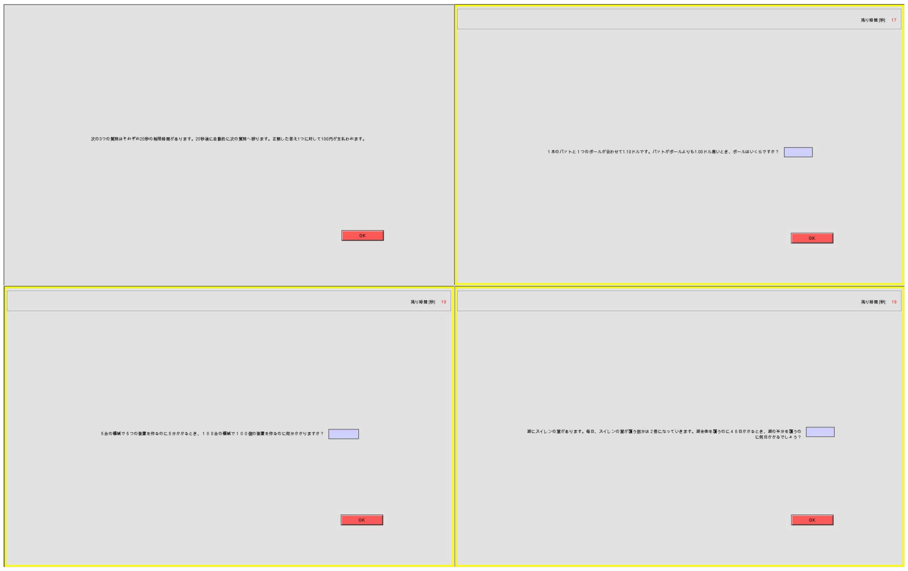

Translation:

Each of the next three questions each has a 20-second time limit. The computer will automatically move to the next question after 20 seconds. You will be paid 100 yen for each correct answer.

A bat and a ball cost $1.10 in total. The bat costs $1.00 more than the ball. How much does the ball cost?

If it takes 5 machines 5 minutes to make 5 widgets, how long would it take 100 machines to make 100 widgets?

In a lake, there is a patch of lily pads. Every day, the patch doubles in size. If it takes 48 days for the patch to cover the entire lake, how long would it take for the patch to cover half of the lake?

Fig 11. zTree screen for dictator game.

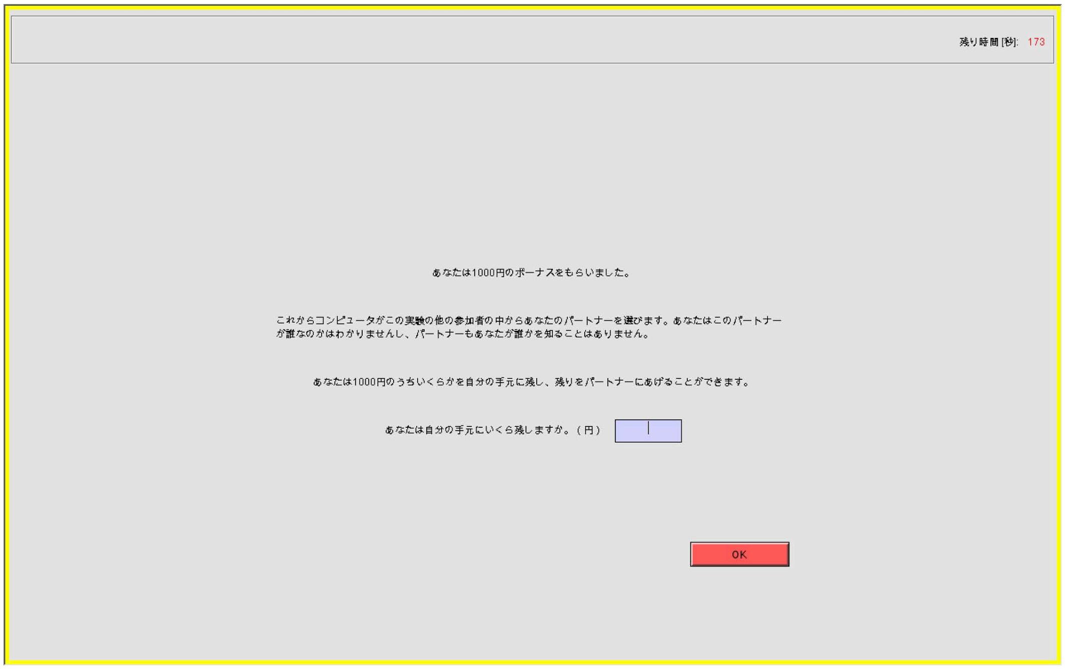

Translation:

You have received a 1000 yen bonus.

The computer will now pick your partner among the other participants in this experiment. You will not know who this partner is, nor will your partner know who you are.

You can keep any amount of the 1000 yen for yourself, and give the rest to your partner.

How much would you like to keep? (yen)

Fig 12. zTree screen for beauty-contest game.

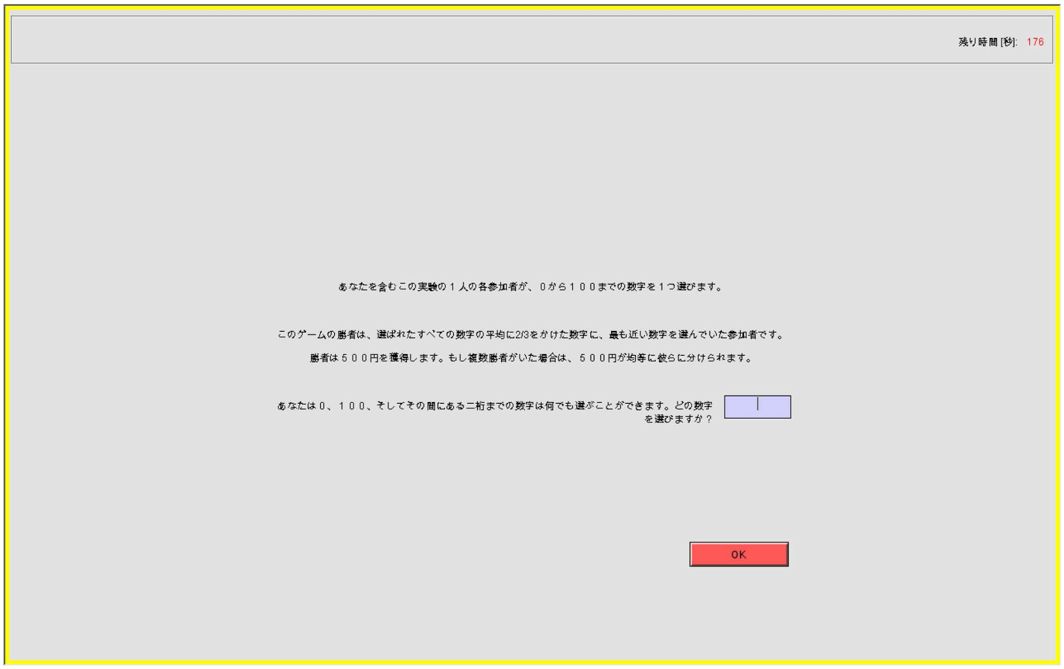

Translation:

Each of the X participants in this experiment (including you) is asked to choose a number between 0 and 100.

The winner of this game is the participant whose number is the closest to 2/3 of the average of all the chosen numbers.

The winner receives 500 yen. In case there is a tie, the amount is going to be split evenly among the winners.

You are allowed to choose 0, 100, and any number in between with up to two digits. Which number do you choose?

Fig 13. zTree screen for risk attitude elicitation through lottery choices.

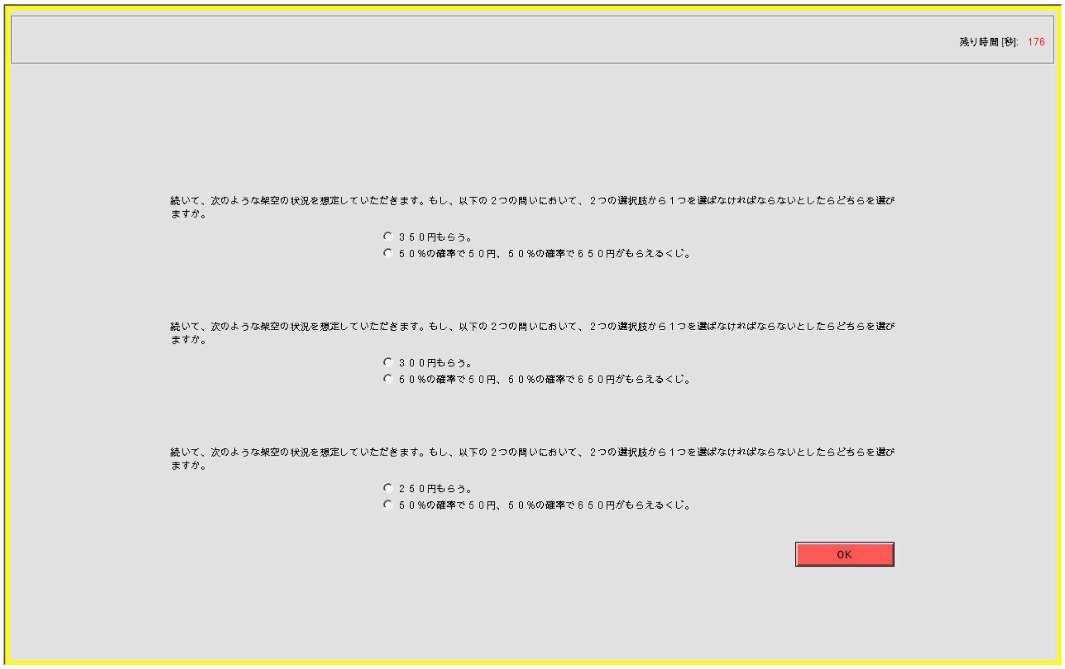

Translation:

Now, imagine the following situation. If you have the choice between them, which of the two options below would you rather have?

1. 350 yen.
2. A lottery giving you a 50% chance of 50 yen and a 50% chance of 650 yen.

Now, imagine the following situation. If you have the choice between them, which of the two options below would you rather have?

1. 300 yen.
2. A lottery giving you a 50% chance of 50 yen and a 50% chance of 650 yen.

Now, imagine the following situation. If you have the choice between them, which of the two options below would you rather have?

1. 250 yen.
2. A lottery giving you a 50% chance of 50 yen and a 50% chance of 650 yen.

Fig 14. zTree screen for risk attitude elicitation through self declaration.

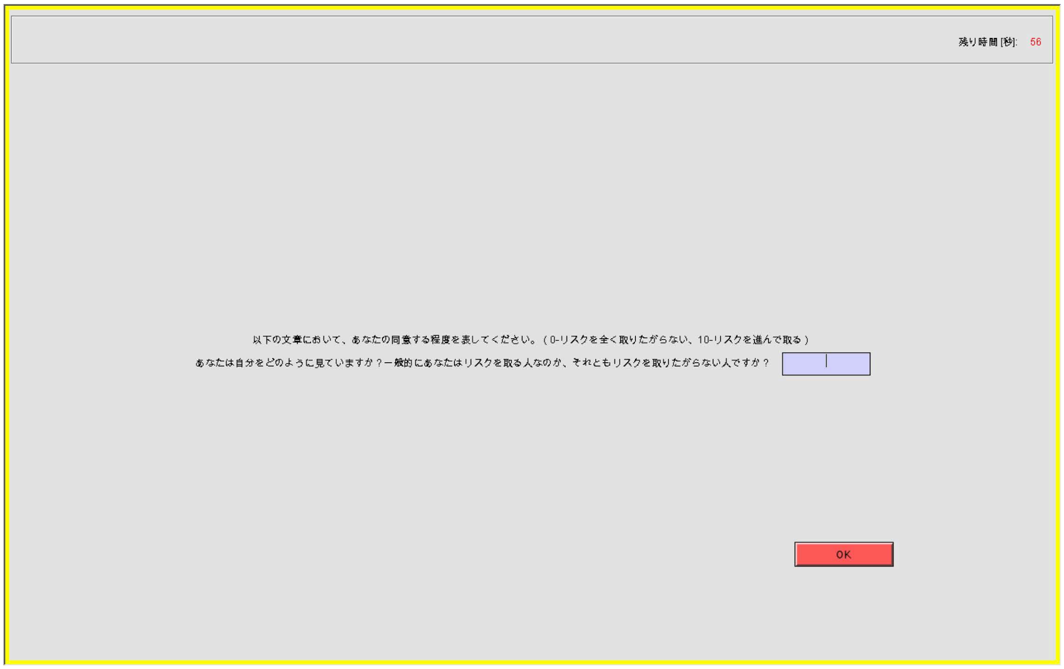

Translation:

For answering the question below, please use a scale from 0 to 10 (0 - “completely unwilling to take risks”, 10 - “very willing to take risks”).

How do you see yourself: are you a person who is generally willing to take risks, or do you try to avoid taking risks?

Fig 15. zTree screen for risk attitude elicitation through bomb risk-elicitation task.

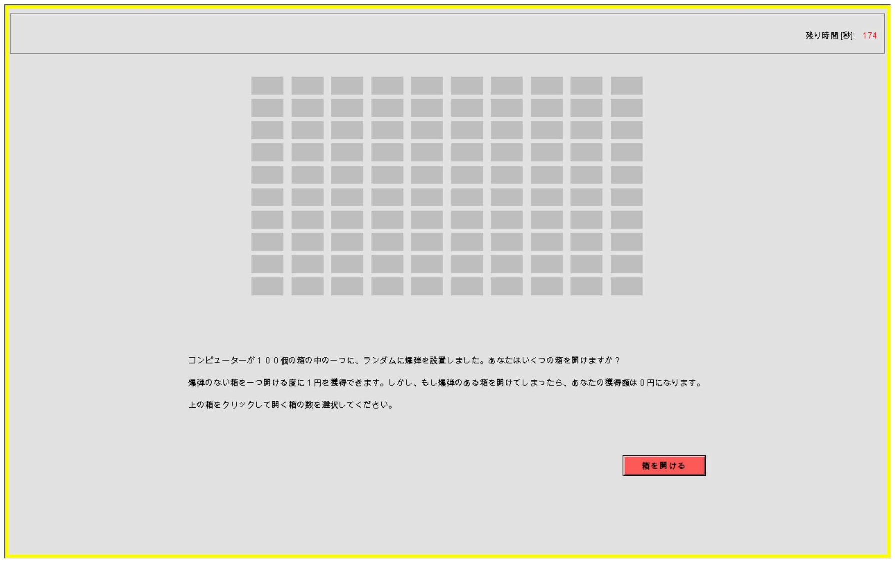

Translation:

The computer has randomly placed one bomb in 1 of 100 boxes. How many boxes will you open?

You earn 1 yen for every box opened. But if you open the box with the bomb, you will earn 0.

Click on the boxes above to select the number of boxes to open.

Fig 16. zTree screen for gift-exchange game.

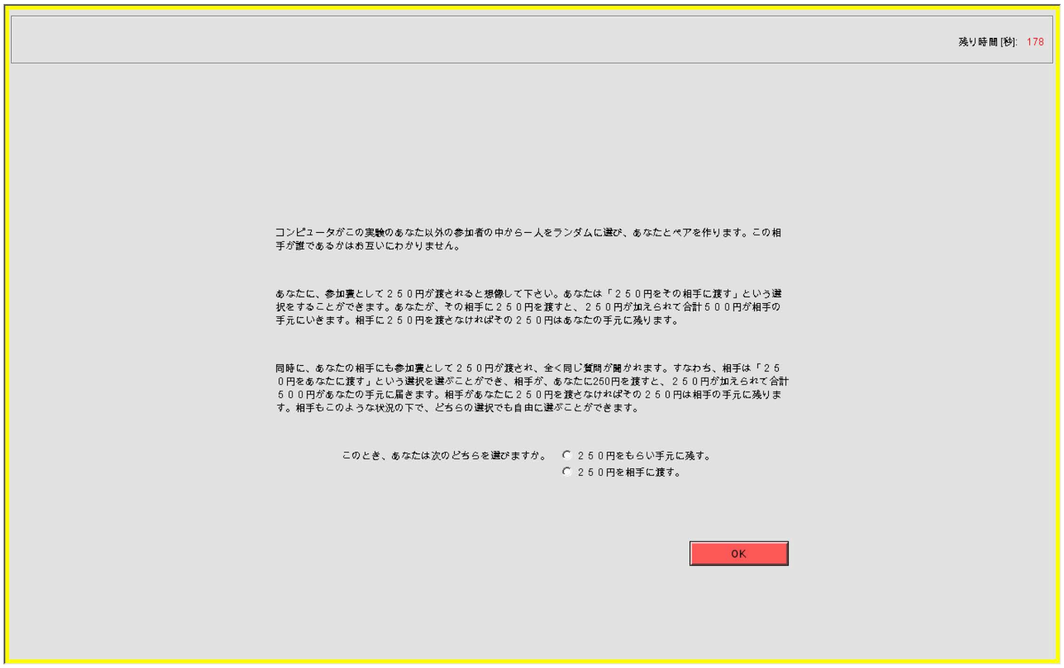

Translation:

The computer will randomly pick one of the other participants in this experiment to form a pair with you. You will not know who this participant is.

Imagine that you are given 250 yen. You have the option of giving 250 yen to the other participant. If you give 250 yen to the other participant, another 250 yen will be added and the other participants will receive a total of 500 yen. If you do not give 250 yen to the other participant, the 250 yen will remain yours.

At the same time, 250 yen will be given to the other participant as well, and the exact same question will be asked. That is, the other participant can choose to give 250 yen to you, and when the other party gives you 250 yen, another 250 yen will be added and you will receive a total of 500 yen. If the other person does not give you 250 yen, the 250 yen will remain hers. The other party is free to choose either option.
Now, which of the following options do you choose?

1. Keep the 250 yen.

2. Give 250 yen to the other participant.

Fig 17. zTree screen for joy-of-destruction game.

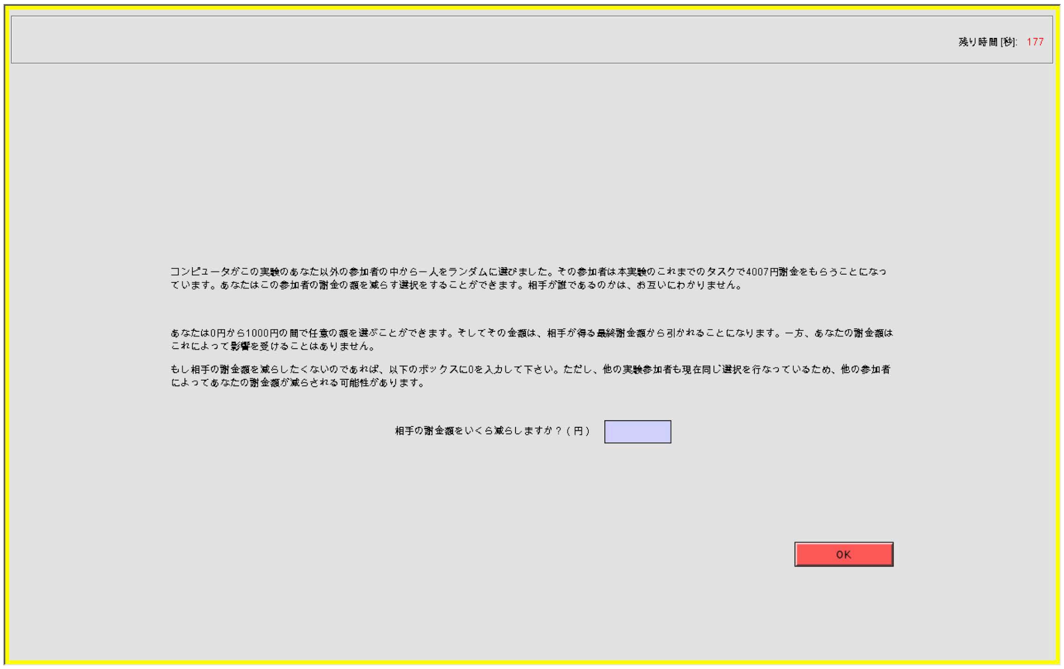

Translation:

The computer has randomly picked one of the other participants in this experiment. That participant is about to receive a total of XXXX yen as earning from the tasks so far in this experiment. You can choose to reduce the amount of this participant's award. You will not know who this participant is.

You can choose any amount between 0 and 1000 yen, and that amount will be deducted from the final earnings that the other participant gets. At the same time, your earnings will not be affected by this.

If you do not want to reduce the other participant's earning, enter 0 in the box below. However, other participants are currently making the same choices, so an other participant may reduce your award.
By how much would you like to reduce the other participant's earning? (yen)

Debriefing at the end of the experiment

This is the end of the experiment. Thank you.

We owe you an apology about the nature of our experiment. The committee members who supervised your group activity intentionally took a harsh attitude in order to increase your stress level. We would like to apologize for the distress that this caused. We did not videotape your performance, and your performance will not be evaluated by a professor at Waseda University. The method that we followed is one of the standard methods used in the area of psychology, and we have obtained an approval for our research from the Ethics Committee on Human Subjects of Waseda University. Your understanding is greatly appreciated.

We will have more rounds of this experiment. If somebody asks you what you did in our experiment, you may answer that it was about decision-makings under stress, but please do not reveal the details of the experiment.
